# Stress–strain curve and elastic behavior of the fibrotic lung with usual interstitial pneumonia pattern during protective mechanical ventilation

**DOI:** 10.1038/s41598-024-63670-z

**Published:** 2024-06-07

**Authors:** Roberto Tonelli, Raffaella Rizzoni, Salvatore Grasso, Andrea Cortegiani, Lorenzo Ball, Anna Valeria Samarelli, Riccardo Fantini, Giulia Bruzzi, Luca Tabbì, Stefania Cerri, Linda Manicardi, Dario Andrisani, Filippo Gozzi, Ivana Castaniere, Marry R. Smit, Frederique Paulus, Lieuwe D. J. Bos, Enrico Clini, Alessandro Marchioni

**Affiliations:** 1 Respiratory Diseases Unit, Department of Medical and Surgical Sciences, University Hospital of Modena, University of Modena Reggio Emilia, Modena, Italy; 2 Laboratory of Cell Therapies and Respiratory Medicine, Department of Medical and Surgical Sciences for Children and Adults, University Hospital of Modena, Modena, Italy; 3 Department of Engineering, University of Ferrara, via Saragat 1, Ferrara, Italy; 4 Dipartimento di Medicina di Precisione e Rigenerativa e Area Ionica (DiMePre-J) Sezione di Anestesiologia e Rianimazione, Università degli Studi di Bari “Aldo Moro”, Ospedale Policlinico, Bari, Italy; 5 Department of Surgical, Oncological and Oral Science (Di.Chir.On.S.), University of Palermo, Palermo, Italy; 6 Department of Anesthesia, Intensive Care and Emergency, Policlinico Paolo Giaccone, Palermo, Italy; 7 Department of Surgical Sciences and Integrated Diagnostics, University of Genoa, Genoa, Italy; 8 Department of Intensive Care, Amsterdam University Medical Centers, University of Amsterdam, Amsterdam, The Netherlands

**Keywords:** Interstitial lung disease, Idiopathic pulmonary fibrosis, Lung fibrosis, Stress, Strain, Lung elastance, Specific elastance, Usual interstitial pneumonia, Acute respiratory failure, Acute respiratory distress syndrome, Respiratory mechanics, End-inspiratory transpulmonary pressure, End-expiratory transpulmonary pressure, Invasive mechanical ventilation, Ventilator-induced lung injury, Transpulmonary pressure, Medical research, Respiratory tract diseases, Respiratory distress syndrome

## Abstract

Patients with acute exacerbation of lung fibrosis with usual interstitial pneumonia (EUIP) pattern are at increased risk for ventilator-induced lung injury (VILI) and mortality when exposed to mechanical ventilation (MV). Yet, lack of a mechanical model describing UIP-lung deformation during MV represents a research gap. Aim of this study was to develop a constitutive mathematical model for UIP-lung deformation during lung protective MV based on the stress–strain behavior and the specific elastance of patients with EUIP as compared to that of acute respiratory distress syndrome (ARDS) and healthy lung. Partitioned lung and chest wall mechanics were assessed for patients with EUIP and primary ARDS (1:1 matched based on body mass index and PaO_2_/FiO_2_ ratio) during a PEEP trial performed within 24 h from intubation. Patient’s stress–strain curve and the lung specific elastance were computed and compared with those of healthy lungs, derived from literature. Respiratory mechanics were used to fit a novel mathematical model of the lung describing mechanical-inflation-induced lung parenchyma deformation, differentiating the contributions of elastin and collagen, the main components of lung extracellular matrix. Five patients with EUIP and 5 matched with primary ARDS were included and analyzed. Global strain was not different at low PEEP between the groups. Overall specific elastance was significantly higher in EUIP as compared to ARDS (28.9 [22.8–33.2] cmH_2_O versus 11.4 [10.3–14.6] cmH_2_O, respectively). Compared to ARDS and healthy lung, the stress/strain curve of EUIP showed a steeper increase, crossing the VILI threshold stress risk for strain values greater than 0.55. The contribution of elastin was prevalent at lower strains, while the contribution of collagen was prevalent at large strains. The stress/strain curve for collagen showed an upward shift passing from ARDS and healthy lungs to EUIP lungs. During MV, patients with EUIP showed different respiratory mechanics, stress–strain curve and specific elastance as compared to ARDS patients and healthy subjects and may experience VILI even when protective MV is applied. According to our mathematical model of lung deformation during mechanical inflation, the elastic response of UIP-lung is peculiar and different from ARDS. Our data suggest that patients with EUIP experience VILI with ventilatory setting that are lung-protective for patients with ARDS.

## Introduction

Patients with fibrotic interstitial lung disease (ILD) may experience acute worsening of their clinical status leading to severe acute hypoxic respiratory failure that needs respiratory assistance^1^. Although acute exacerbations (AE) of disease represent a major cause of death in patients with idiopathic pulmonary fibrosis (IPF), the elastic properties of the fibrotic lung and its interplay with non-invasive and invasive respiratory support remains largely unknown^2^. What is known from clinical practice is that the tissue architecture of the fibrotic lung with usual interstitial pneumonia (UIP) pattern limits parenchymal deformation and increases the risk of ventilator-induced lung injury (VILI) when mechanical ventilation (MV) is required^3^. In lung physiology, stress is defined as the pressure developed within the lung structures onto which a distending force (namely transpulmonary pressure) is applied whereas strain is the ratio of the change in lung volume to the resting lung volume during lung inflation^4,5^. Lung tissue exhibits a heterogenous nonlinear stress–strain behavior arising from the mechanical interaction between the extra cellular matrix (ECM) constituents, collagen and elastin fibers^6–8^. In previous studies, we hypothesized static strain as a major determinant of VILI for these patients^9^.

Studies focused on early acute respiratory distress syndrome (ARDS) patients, showed that ARDS-lungs exhibit a specific lung elastance that is similar to healthy lungs, suggesting that the elastic properties of the aerated lung regions are not affected by diffuse alveolar damage^10^. ARDS, like IPF, is characterized by a heterogeneous elasticity of the lung parenchyma. However, unlike in IPF, the pathological changes associated with ARDS result from alveolar inflammatory exudation impacting an initially healthy lung and are influenced by gravitational forces^3^. By contrast, UIP represents an intrinsic architectural alteration of the lung structure due to the quantitative and qualitative changes in the collagen and elastin networks in alveolar wall tissue^11^.

Understanding the mechanical behavior of the fibrotic lung during passive lung inflation could help critical care physicians to tailor controlled MV and prevent detrimental stress–strain interaction resulting in VILI^12^. Our aim was twofold: the first explorative aim of this study was to investigate the stress–strain behavior and the specific elastance in a cohort of patients with ILD and UIP pattern undergoing lung protective MV during AE of disease (EUIP), compared it to that of patients with ARDS and healthy lung. The second aim was to propose a novel mathematical model to describe ventilator-induced stress and strain in the fibrotic lung.

## Materials and methods

### Study design

This study constitutes an exploratory analysis of a subset of patients retrospectively enrolled in a registered study protocol (NCT05098717 registered on ClinicalTrial.gov on 10/15/2021), whose respiratory mechanical properties under mechanical ventilation have recently been published by our group in comparison to matched ARDS cases^13^. The present investigation has been designed and conducted in two sequential phases: initially, we have retrospectively collected data of patients with UIP pattern that underwent MV during acute exacerbation of disease and protocolized respiratory mechanics assessment and compared them with data from an ARDS matched cohort.

In the second phase, we have firstly elaborated a new mechanical model of the lung that describes an alveolus as spherical, nonlinear elastic, pressurized shell. The mechanical model is based on three parameters: the elastic constants of elastin and collagen, and the collagen volume fraction. To estimate these parameters, we have fitted the pressure–volume response calculated with the mechanical model to the respiratory mechanics data of our cohorts (ARDS and EUIP patients) and available data in literature on healthy lung. Substituting the estimated values of the three parameters into our mechanical model allows us to predict the contributions of elastin and collagen fibers to ventilator-induced lung deformation and calculate the distributions of the radial and hoop stress components.

### First phase

#### Study setting

The study was carried out at the Respiratory Intensive Care Unit (RICU) of the University Hospital of Modena (Italy) and conducted in accordance with the Ethics Committee “Area Vasta Emilia Nord” approval (registered protocol number 327/2022). Informed consent to participate in the study and to allow their clinical data to be analyzed and published were obtained from participants, when available as appropriate.

#### Study population

Patients with ILD and UIP pattern developing acute hypoxic respiratory failure (AHR) due to acute exacerbation (AE) of disease and consecutively admitted to the RICU of the University Hospital of Modena over the period August 1st, 2016 to July 1th, 2022 were considered eligible for enrollment.

Inclusion criteria were as follows: age > 18 years; previously established diagnosis of ILD with a UIP pattern on a high-resolution computed tomography (HRCT) scan; invasive MV in volume-controlled mode; end-expiratory lung volume assessment. According to our institutional protocol, patients with severe EUIP requiring MV are submitted to a standardized protocol of partitioned respiratory mechanics measurements at three different levels of positive end-expiratory pressure (PEEP), i.e. zero PEEP (ZEEP), PEEP_LOW_ (4–8 cmH_2_O) and esophageal pressure (P_es_)-guided PEEP titration to obtain positive end expiratory transpulmonary pressure (P_L,EE_) (PEEP_TITRATED_).

Patients were excluded if they presented any of the followings: chronic obstructive pulmonary disease; neuromuscular disease; chest wall deformities; missing core data (i.e. data on partitioned respiratory mechanics) at medical record analysis.

EUIP population was then compared with a group of patients with pulmonary ARDS under MV extracted from our dataset, treated between 2016 and 2022. Body mass index (BMI) and PaO_2_/FIO_2_ used for matching the EUIP and the pulmonary ARDS groups were those measured at the time of RICU admission.

The stress/strain curve of both populations was compared to that of patients with healthy lung derived from data published by D’Angelo et al.^14^ and Levy et al.^15^, denoted as DA and L data.

#### Data collection

Medical reports, electronic charts and available clinical and physiological datasets were investigated to collect data on demographics, clinical characteristics, arterial blood gases, PaO_2_/FiO_2_ ratio, clinical severity (as assessed by the APACHE II score on RICU admission), standardized partitioned respiratory mechanics assessment and end-expiratory lung volume measurements at each PEEP level.

#### Measurements

On admission, patients were ventilated with a tidal volume of 6 ml/kg of predicted body weight (PBW) computed as in Brower et al.^16^ and with a ventilation strategy aimed at keeping plateau pressure below 25 cmH_2_O. According to our local protocol, sedation was achieved with a midazolam-propofol-remifentanil infusion to obtain a bispectral index between 40 and 60. Patients received cisatracurium to obtain myorelaxation. All subjects were placed in a 30° head up position. PEEP was initially set between 5 and 8 cmH_2_O. All patients underwent esophageal manometry (NutriVent™, nasogastric polyfunctional catheter; SIDAM, Mirandola, Italy), according to standard procedures^17^.

According to our local protocol, respiratory mechanics were further assessed in supine position within 24 h from admission in three consecutive phases at different PEEP levels.

- In the first phase (ZEEP phase), measurements of static respiratory mechanics were performed at ZEEP.
- In the second phase (PEEP_LOW_ phase), static respiratory mechanics were assessed with PEEP set between 4 and 8 cmH_2_O.
- In the third phase (PEEP_TITRATED_ phase), PEEP was titrated in order to achieve a P_L,EE_ > 0 cmH_2_O and according to oxygenation^18^.

At each phase, PEEP level was maintained for 30 min before recording all respiratory parameters and blood withdrawal for blood gas analysis. Respiratory mechanics were performed using the end-inspiratory occlusion technique during constant flow inflation and the end-expiratory occlusion method^19^.

The mechanical variables were measured by means of the respirator’s (Engstrom Carestation [GE Healthcare, USA]) software for respiratory mechanics assessment and the Optivent™ (SIDAM, Mirandola Italy) monitor and computed according to the formulas reported in the Supplementary^5,10,20–22^.

### Second phase

#### Mechanical model development

**Figure 1:**
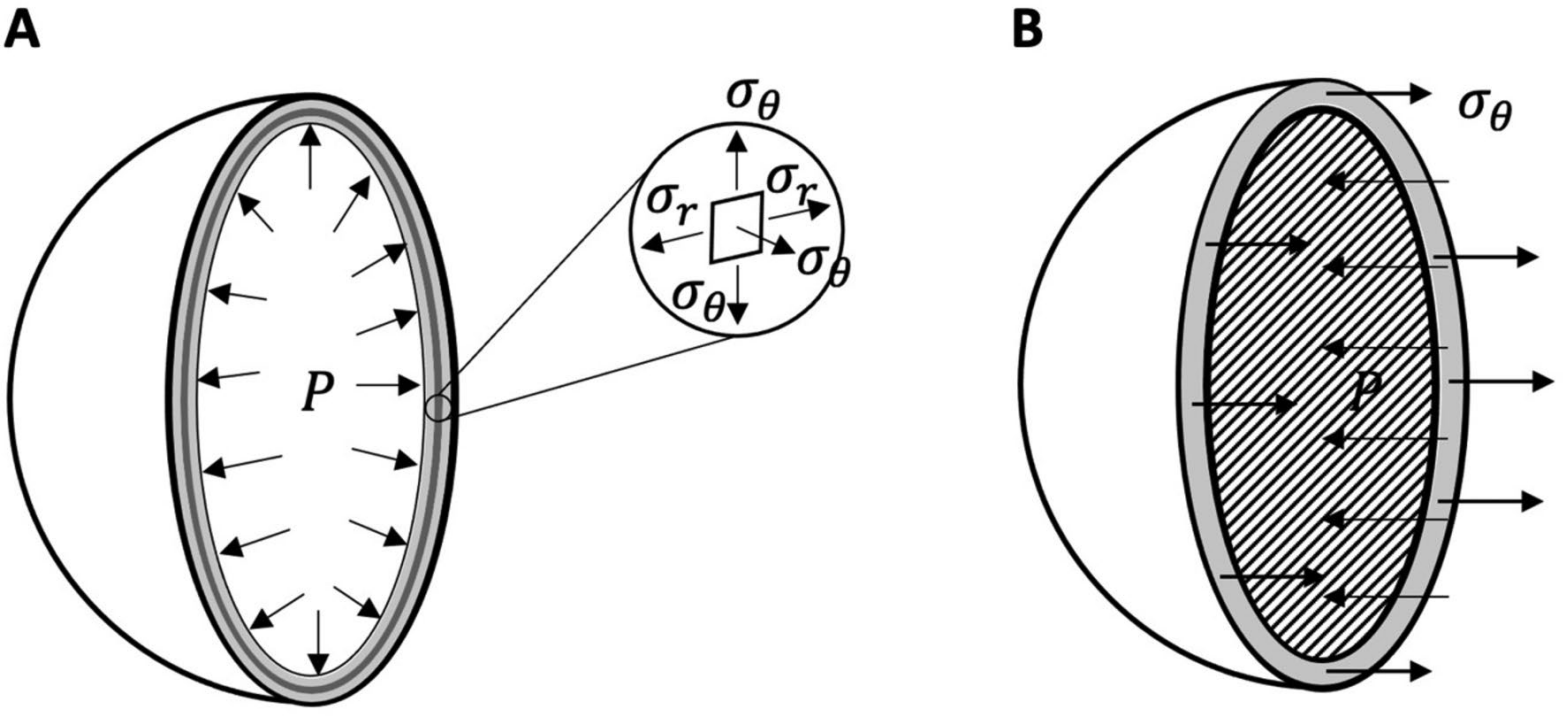
(**Panel A**) geometry of the three-layered shell modeling an alveolus. Dark gray indicates a layer of collagen interposed between two layers composed of elastin and ground substance, colored in light gray. The shell is inflated by an inner pressure $P.$ The inset shows the stress components acting at each point of the shell material: the radial stress component, ${\sigma }_{r}$, is in the radial direction, and the circumferential (or hoop) stress components, ${\sigma }_{\theta }$ and ${\sigma }_{\varphi }$ (here ${\sigma }_{\varphi }={\sigma }_{\theta },$ due to axial symmetry), are in the tangential direction of the meridians and the parallels of the sphere. (**Panel B**) For a homogeneous shell, force equilibrium requires the resultant of ${\sigma }_{\theta },$ acting on the small area depicted in gray, to be much larger than the resultant of the pressure $P$ acting on the dashed larger area.

We propose a computational model for the lung based on the mechanical behavior of a single alveolus. Alveolar models and their generalizations have been successfully proposed to explain the pressure–volume response of the normal lung^22–24^. Specifically, they allow to relate the macroscopic response of the lung (the pressure–volume curve) and the mechanical behavior of the alveolus. This is useful to assess the stresses and local elastance in the alveolar walls to investigate the risk of overdistension and epithelial cell damage when the macroscopic strain is at safe values. In our approach, the alveolus is modeled as an inflated thin-walled hollow spherical shell schematically shown in Fig. 1 The shell is initially stress-free, and it is subject to the internal pressure $P$. As shown in previous studies^22–24^ elastin and collagen dominate the elastic behavior of the alveolar tissue, the contribution of interstitial cells being negligible^25–27^. It is well known that collagen and elastin fibers form an interlinked random mesh in lung parenchyma^28,29^. In our alveolar model, the microstructure of the two types of fibers is smeared out, as usual in homogenization approaches^30,31^. In other words, the detail of the microstructure is spatially regularized, implying that the network of elastin and collagen fibers forming the alveolar space are modeled as spherical layers of equivalent homogeneous materials. Accordingly, we assume that the shell is composed of alternating layers of possible dissimilar thickness, as shown in Fig. 1. Each layer is composed of a different nonlinear hyper-elastic, isotropic and incompressible material, modelling a different fiber contribution. Even though the analysis is performed for any number of layers, for computational testing we implement only three layers, two external ones composed of elastin fibers and ground substance and an inner layer modeling collagen fibers and their interaction with elastin. The responses in traction of the materials composing the two types of layers are specified by the strain energy functions in Eqs. (16, 17) Supplementary^21,22,32–38^. Strain energy functions of this type have been experimentally validated via uniaxial tension tests on living precision-cut rat lung slices^34,35^. In addition, it can be easily verified that the stress–strain curve associated to the strain energy function for collagen (Eq. 17) has a vanishing slope at the origin followed by a steep ascending branch, mimicking the initial flaccidness and the progressive recruitment of collagen fibers as strain increases^32,36–38^.

The model focuses on the elastic effects on mechanical fields for static inflation, so viscoelasticity is not included in the present analysis.

The development of the mechanical model was carried out according to the following steps:

1. First, we have mathematically assessed the equilibrium state of a multilayered pressurized sphere and deduced the corresponding pressure–volume response (see Eq. (13), Supplementary). This relation has been specialized to the case of three layers composed of nonlinear elastic materials, whose behavior is described by strain energies functions (see Eqs. (16, 17, 18), Supplementary). For a shell geometry with a ratio of thickness over inner radius equal to 0.05^22^, the number of model parameters entering the pressure–volume response (see Eq. (18), Supplementary) is just three: the elastic constants of elastin and collagen, c_1_ and c_2_, and the volume fraction of collagen, ${\chi }_{2}$.
2. The pressure–volume relationship (see Eq. (18), Supplementary) was then fitted to the patients’ data. Even though at the individual level, the data collected on EUIP and ARDS patients show some differences, we focused on the average behavior of patients with either EUIP or ARDS. For this reason, we performed a single fitting with all available data for each disease. The fitting allowed us to estimate the three model parameters for each patient population.
3. Finally, using the estimated values of the model parameters, we evaluated the different contributions of collagen and elastin to ventilator-induced lung deformation, and we assessed the piecewise distribution of radial and hoop stress along the thickness of the shell model (see Eqs. (24, 29) in Supplementary). The calculation of the radial and hoop stress components at different pressure and strain levels provides further insight on the different stress level inside the two types of fibers (elastin and collagen).

The alveolar model proposed in the present paper is analytical, leading to an exact and thus computationally fast pressure–volume relationship (Eq. 18, Supplementary).

### Analysis plan

Data were displayed as median and IQR (interquartile range) for continuous variables and numbers and percentages for dichotomous variables. The paired Student’s *t*-test was used to assess the difference between variables measured at different PEEP levels, when distributed normally; otherwise, the Wilcoxon test was used. As an exploratory analysis, we compared the mechanical variables of EUIP patients with an ARDS population extracted from our dataset, between 2016 and 2022. The ARDS comparison cohort was built using a case–control matching procedure according to BMI and PaO_2_/FiO_2_ ratio. Comparison between continuous variables was performed with Student’s *t*-test when distributed normally; otherwise, the Wilcoxon test was used. Dichotomous variables were compared using the χ^2^ test or Fisher's exact test, where appropriate. ANOVA and Kruskal–Wallis were used to test an interaction for whether the change in strain according to PEEP levels were different between groups.

For normal lung, the model was fit to inflationary static pressure–volume data from literature (D’Angelo et al.^14^ and Levy et al.^15^). In particular, Fig. 7 (left, single patient) in D’Angelo et al.^14^ and Fig. 5 in Levy et al.15 were used to extract the data. Data were digitized by means of WebPlotDigitizer ( https://automeris.io/WebPlotDigitizer, version 4.6, Pacifica, California, USA). To generate the stress–strain data, a functional residual capacity equal to 0.57 l was assumed for both sets of original data. In the absence of values for pleural pressure in D’Angelo et al.^14^ and Levy et al.^15^, static pressure was assumed equal to end-inspiratory transpulmonary pressure. After having generated the stress–strain data for normal lungs, the curve fitting toolbox in MATLAB R2023a (MathWorks, CA, USA) was used to fit the stress–strain model given by Eq. (18) (Supplementary). Analogous fitting was performed for the stress–strain data collected on EUIP and ARDS. Further, the stress–strain curve was graphed, and a partitioned analysis of the secant (specific elastance) and tangent modulus was performed to study the curves behavior. The isolated global and partitioned contributions of elastin and collagen were further investigated according to the constitutive model and considering data on multiscale modeling of collagen and elastin proteins available in literature^26,34,36,38,40^. Statistics was performed using SPSS version 25.0 with PSMATCHING3 R Extension command (IBM Corp., Armonk, NY, USA) and GraphPad Prism version 8.0 (GraphPad Software, Inc., La Jolla, Ca, USA), unless otherwise indicated.

## Results

### Clinical features and respiratory mechanics of study population

The flowchart of this study is shown in eFigure 1, Supplementary. Over the study period a total of 21 patients with EUIP resulted eligible for enrollment. Of these, 5 patients were analyzed according to inclusion criteria. All of them showed a definite UIP pattern on HRCT scan. Patients were prevalently male (n = 4) with a median age of 62 years (56–68) (eTable 1, Supplementary). Two of them were diagnosed with IPF. Among the others, one had rheumatoid arthritis associated ILD, one had chronic hypersensitivity pneumonitis and one presented an undetermined ILD. All patients died as inpatients while on MV. EUIP and matched ARDS groups were similar for clinical severity scores (APACHE and SAPS II, eTable 1, Supplementary). All ARDS patients presented a pulmonary ARDS occurring from lung infection.

Respiratory mechanics of EUIP and matched ARDS groups at different PEEP levels are showed in Table 1. The PEEP_LOW_ level was similar, whereas PEEP_TITRATED_ resulted in higher PEEP level in the ARDS group (Table 1).

**Table 1:** Blood gas analyses and respiratory mechanics of the EUIP and the ARDS population at different PEEP levels

Variable	EUIP	ARDS	*p*-value
ZEEP phase			
E_L_, cmH_2_O/L	43.2 (39.9–54.4)	21 (17.3–35.6)	0.01
E_cw_, cmH_2_O/L	3.3 (2.8–7)	4.5 (4–6.3)	0.72
E_tot_, cmH_2_O/L	50 (43.8–57.4)	26.4 (21.6–41.2)	0.01
P_L,EI_, cmH_2_O	16.1 (13.3–18.5)	7 (4.9–7)	0.0002
P_L,EE_, cmH_2_O	− 4.3 (− 7.7-− 2.9)	− 4 (− 7.5-− 1.2)	0.71
ΔP_aw_, cmH_2_O	17.5 (16.3–19.3)	21.2 (17.5–22.8)	0.13
ΔP_L_, cmH_2_O	21.3 (18.1–23.8)	9.4 (8.9–13.2)	0.0003
EELV, ml	693 (588–717)	820 (599–1047)	0.18
Vt/PBW, ml/kg	4.9 (3.9–5.6)	5.1 (4.6–5.4)	0.67
Vt, ml	360 (315–414)	408 (396–426)	0.09
Strain, ratio	0.58 (0.49–0.62)	0.5 (0.39–0.67)	0.68
pH, value	7.42 (7.39–7.44)	7.39 (7.37–7.41)	0.17
pO2, cmH_2_O	75 (72–80)	80 (65–90)	0.73
pCO2, cmH_2_O	38 (35–41)	39 (38–42)	0.35
PEEP_LOW_ phase			
E_L_, cmH_2_O/L	44 (38.8–57.8)	18.3 (15.4–29.5)	0.003
E_cw_, cmH_2_O/L	3.6 (2.7–7)	4.3 (4–6.4)	0.73
E_tot_, cmH_2_O/L	52 (42.3–60.9)	23.6 (19.5–35.3)	0.005
P_L,EI_, cmH_2_O	14 (10.9–18.8)	12 (8.7–15)	0.26
P_L,EE_, cmH_2_O	− 2.2 (− 6.5-− 1)	− 2.1 (− 5.4-− 0.6)	0.78
ΔP_aw_, cmH_2_O	18.2 (14.8–18.8)	20.8 (16.2–26)	0.18
ΔP_L_, cmH_2_O	18.4 (15.6–21.8)	16.1 (9.3–19.4)	0.32
PEEP, cmH_2_O	4 (4–4)	4 (4–5)	0.71
EELV, ml	702 (625–762)	1010 (733–1175)	0.04
Vt/PBW, ml/kg	5.3 (4.4–6.1)	5.8 (5.4–6.4)	0.27
Vt, ml	401 (354–442)	476 (471–499)	0.01
Strain, ratio	0.62 (0.6–0.74)	0.96 (0.63–1.1)	0.08
pH, value	7.4 (7.38–7.43)	7.39 (7.35–7.4)	0.18
pO_2_, cmH_2_O	75 (72–78.5)	80 (69.5–95.5)	0.31
pCO_2_, cmH_2_O	39 (36–42.5)	40 (38.5–44)	0.39
PEEP_TITRATED_ phase			
E_L_, cmH_2_O/L	50 (42.8–60.5)	15.4 (14.4–20.9)	< 0.0001
E_cw_, cmH_2_O/L	3.9 (3.4–7.2)	4.3 (4–6.5)	0.97
E_tot_, cmH_2_O/L	59 (47.3–64.2)	21 (18.6–26.6)	< 0.0001
P_L,EI_, cmH_2_O	26.2 (19.9–29.8)	18.2 (16.5–20.3)	0.03
P_L,EE_, cmH_2_O	0.3 (0.3–1.4)	0.6 (0.1–1)	0.63
ΔP_aw_, cmH_2_O	20 (17.3–21.8)	16.5 (14–18.4)	0.06
ΔP_L_, cmH_2_O	25 (19–29.5)	17.6 (15.6–20.2)	0.04
PEEP, cmH_2_O	12 (10–14)	14 (12–17.5)	0.03
EELV, ml	714 (635–755)	1260 (912–1575)	0.01
Vt/PBW, ml/kg	5.6 (4.9–6.7)	6.5 (6.3–6.7)	0.12
Vt, ml	438 (388–486)	540 (505–599)	0.01
Strain, ratio	0.69 (0.63–0.83)	1.8 (1.5–1.9)	< 0.0001
pH, value	7.39 (7.36–7.42)	7.36 (7.32–7.39)	0.14
pO2, cmH_2_O	78 (55–85)	80 (73–91)	0.3
pCO2, cmH_2_O	40 (37–44)	42 (40–48)	0.27

Data are presented as median value and IQR. *EUIP, Acute exacerbation of interstitial lung disease with usual interstitial pneumonia pattern; ARDS, Acute respiratory distress syndrome; IQR, Interquartile range;* ΔP_L_, Transpulmonary driving pressure; P_L,EI_, End-inspiratory transpulmonary pressure; P_L,EE_, End-expiratory transpulmonary pressure; ΔP_aw_, Driving pressure; E_tot_, Respiratory system elastance; E_cw_, Chest wall elastance; E_L_, Lung elastance;* PEEP, Positive end-expiratory pressure; EELV, End-expiratory lung volume; PBW Predicted body weight.*

At ZEEP, EUIP patients had higher E_L_ (43.2 [39.9–54.4] cmH_2_O/L, *p* = 0.01) as compared to ARDS patients (Table 1). During the PEEP_LOW_ and the PEEP_TITRATED_ phases, EUIP patients had higher E_L_ (44 cmH_2_O/L vs. 18.3 cmH_2_O/L, *p* = 0.003 and 50 cmH_2_O/L vs. 15.4 cmH_2_O/L, *p* = 0.0001, respectively) and lower end-expiratory lung volume (702 ml vs. 1010 ml, *p* = 0.04 and 714 ml vs. 1260 ml, *p* = 0.01, respectively) as compared to ARDS (Table 1). Strain was not different at ZEEP and PEEP_LOW_ between the groups (0.58 vs. 0.5 cmH_2_O/L, *p* = 0.68 and 0.62 vs. 0.96, *p* = 0.08, respectively), while EUIP patients showed significantly lower strain values during the PEEP_TITRATED_ phases (0.62 vs. 1.8, *p* < 0.0001). Whereas EUIP and ARDS patients presented an increase in strain during the PEEP_TITRATED_ phase as compared to both ZEEP and PEEP_LOW_ phases, the amount of change resulted significantly higher for the latter group (*p* = 0.003, eFigure 2, Supplementary).

**Figure 2:**
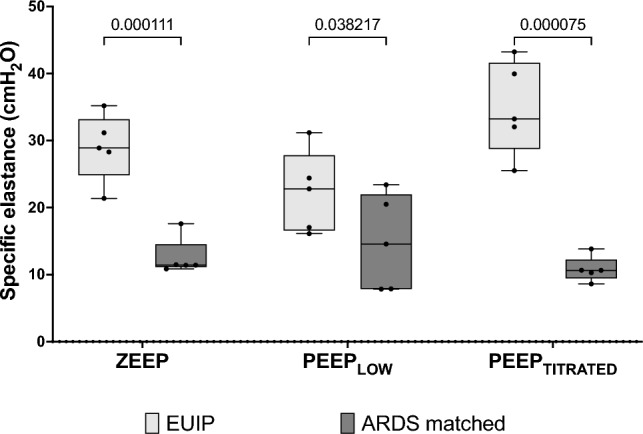
Measured individual values of specific elastance of EUIP and ARDS patients at each PEEP level. For EUIP patients specific elastance was 28.9 (24.8 – 33.2) cmH_2_O, 22.8 (16.6–27.8) cmH_2_O and 33.2 (28.8–41.6) cmH_2_O at ZEEP, PEEP_LOW_ and PEEP_TITRATED_ respectively. For ARDS patients specific elastance was 11.4 (11.1–14.5) cmH_2_O, 14.6 (7.9–22) cmH_2_O and 10.6 (9.5–12.2) cmH_2_O respectively. Specific elastance was different among group at each PEEP level. EUIP, Acute exacerbation of interstitial lung disease with usual interstitial pneumonia pattern; ARDS, Acute respiratory distress syndrome.

The overall specific elastance was notably elevated in EUIP compared to ARDS (28.9 [22.8–33.2] cmH_2_O versus 11.4 [10.3–14.5] cmH_2_O, respectively), and this trend persisted across all PEEP levels (Fig. 2).

### Stress/strain curve and model fitting

The pressure–volume response calculated with the mechanical model was fitted to data taken from the literature for healthy patients and to the collected data on EUIP and ARDS patients. For the shell geometry, we followed Jawde et al. in choosing a ratio of thickness over inner ratio equal to 0.05^22,41^. Table 2 shows the fitting results for the three model parameters: the elastic constant of elastin, ${c}_{1},$ the elastic constant of collagen, ${c}_{2}$, and the collagen thickness fraction ${\chi }_{2}.$ The quality of fitting is given by the adjusted R-square values reported in Table 2, that also shows the confidence intervals for the three parameters. The estimates indicate a range of variation 5.2–18.5 kPa for the elastic parameter ${c}_{1}$ of elastin, and of 35.8–68.8 kPa for the elastic parameter ${c}_{2}$ of collagen.

**Table 2:** Fitting results for the elasticity parameters, ${c}_{1}$ and ${c}_{2}$, and the collagen thickness fraction ${\chi }_{2}$ in the shell model.

Data source	Adj-R^2^	SSE	Elastin and ground substance	Collagen	
			${c}_{1}$*(cmH*_*2*_*O)*	${c}_{2}$*(cmH*_*2*_*O)*	${\chi }_{2}$*(-)*
Healthy lung (D’Angelo et al.^14^)	0.9774	2.458	53.29 [39.71–66.87]	365.0 [246–483.9]	0.05 [− 0.19–0.29]
Healthy lung (Levy et al.^15^)	0.9754	8.07	74.64 [66.21–83.06]	392.9 [315.7–470.0]	0.04 [− 0.07–0.15]
ARDS	0.7435	135.0	95.0 [70.0–120.0]	212.0 [91.05–333.0]	0.08 [− 0.16–0.32]
EUIP	0.5743	340.9	189.4 [102.1–276.7]	702.3 [370.7–1034]	0.31 [− 0.01–0.63]

The fitting has been obtained using Eq. (18) for human pressure–volume curves for normal, ARDS and EUIP lungs. The thickness fraction of collagen, ${\chi }_{2},$ has been found about 4% in normal lungs and in ARDS patients, and 31% in EUIP patients. The volume fraction of elastin and ground material is simply given by ${1-\chi }_{2}$.The 95% confidence intervals are indicated in square brackets. Data are presented as median and IQR.

EUIP, Acute exacerbation of interstitial lung disease with usual interstitial pneumonia pattern; ARDS, Acute respiratory distress syndrome; IQR, Interquartile range.

The lower values of ${c}_{1}$ and ${c}_{2}$ in Table 2, estimated for healthy human lungs, are comparable with those reported by Rausch and coworkers, ${c}_{1}$= 4.1 kPa and ${c}_{2}$= 20.7 kPa, obtained by fitting experimental data of uniaxial tension tests on living precision-cut rat lung slices^34^. The values estimated for ${\chi }_{2}$ in Table 2 for normal and ARDS lungs agree with the findings of Mercer and Crapo, who report a mean of 7% for the volume fraction of collagen in healthy human lungs far from the alveolar duct^42^. For EUIP patients, Table 2 shows an increase in the elastic parameters ${c}_{1}$ and ${c}_{2}$, as well as an increase in the collagen fraction ${\chi }_{2}.$.

**Figure 3:**
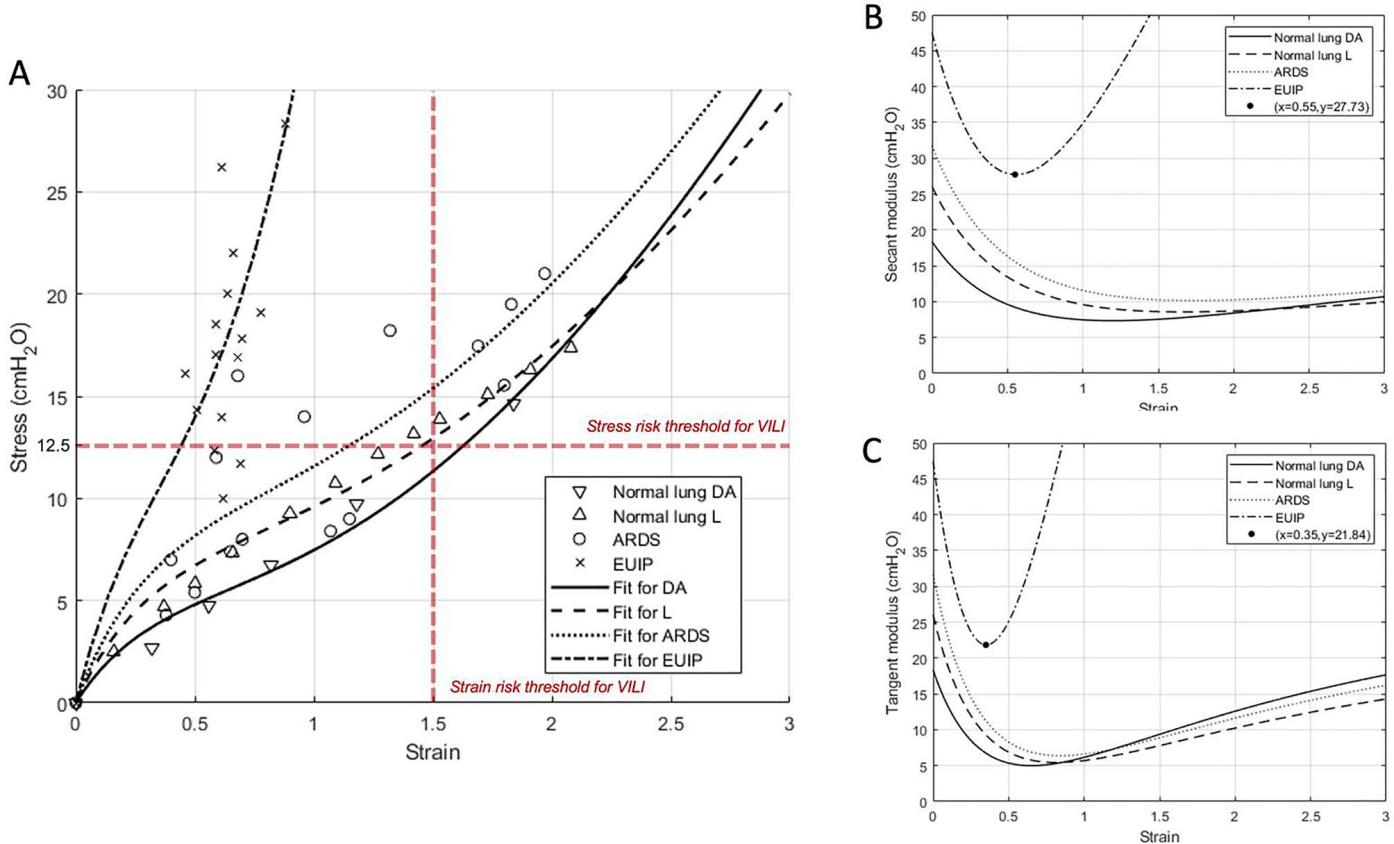
(**Panel A**) Pressure–volume curves for EUIP, ARDS and healthy patients^14,15^ obtained by using Eq. (18). The VILI risk thresholds for stress and strain values derived from ARDS literature are indicated according to Tonetti et al.^47^ and Protti et al.^48^. None of the 5 EUIP patients reached global strain values above 1, irrespective of the PEEP applied. For ARDS patients, the highest stress values were reached for strain levels between 1.5 and 2. The same was described when plotting data in healthy lungs^14,15^. With reference to the mechanical model, strain and stress indicate the relative volume change and the pressure applied inside the composite sphere, respectively. (**Panel B**) Partitioned analysis of the pressure–volume curves for EUIP, ARDS and healthy patients^14,15^ obtained by using Eq. (18) showing the behavior of the secant modulus (specific elastance). When fitting EUIP data, the minimum point was reached for strain = 0.55. (**Panel C**) Partitioned analysis of the pressure–volume curves for EUIP, ARDS and healthy patients^14,15^ obtained by using Eq. (18) illustrating the behavior of the tangent modulus. When fitting EUIP data, the minimum point was reached for strain = 0.35. EUIP, Acute exacerbation of interstitial lung disease with usual interstitial pneumonia pattern; ARDS, Acute respiratory distress syndrome.

The stress/strain curves calculated according to Eq. (18) (Supplementary) for EUIP and ARDS patients and for healthy subjects are illustrated in Fig. 3, panel A. The curves for healthy patients are obtained by fitting the proposed mathematical model to the data taken from references^14,15^. None of the EUIP patients reached global strain above 1. Four out 5 patients with EUIP reached levels of strain above 0.5 that corresponded to stress values higher than 12.5 cmH_2_O, irrespective of the selected PEEP. For ARDS patients and healthy subjects, the highest stress values were reached for strain levels between 1.5 and 2. The partitioned analysis of the secant and tangent modulus of EUIP patients showed that the minimum point was reached for strain value of 0.55 (Fig. 3, panel B) and 0.35 (Fig. 3, panel C) respectively.

**Figure 4:**
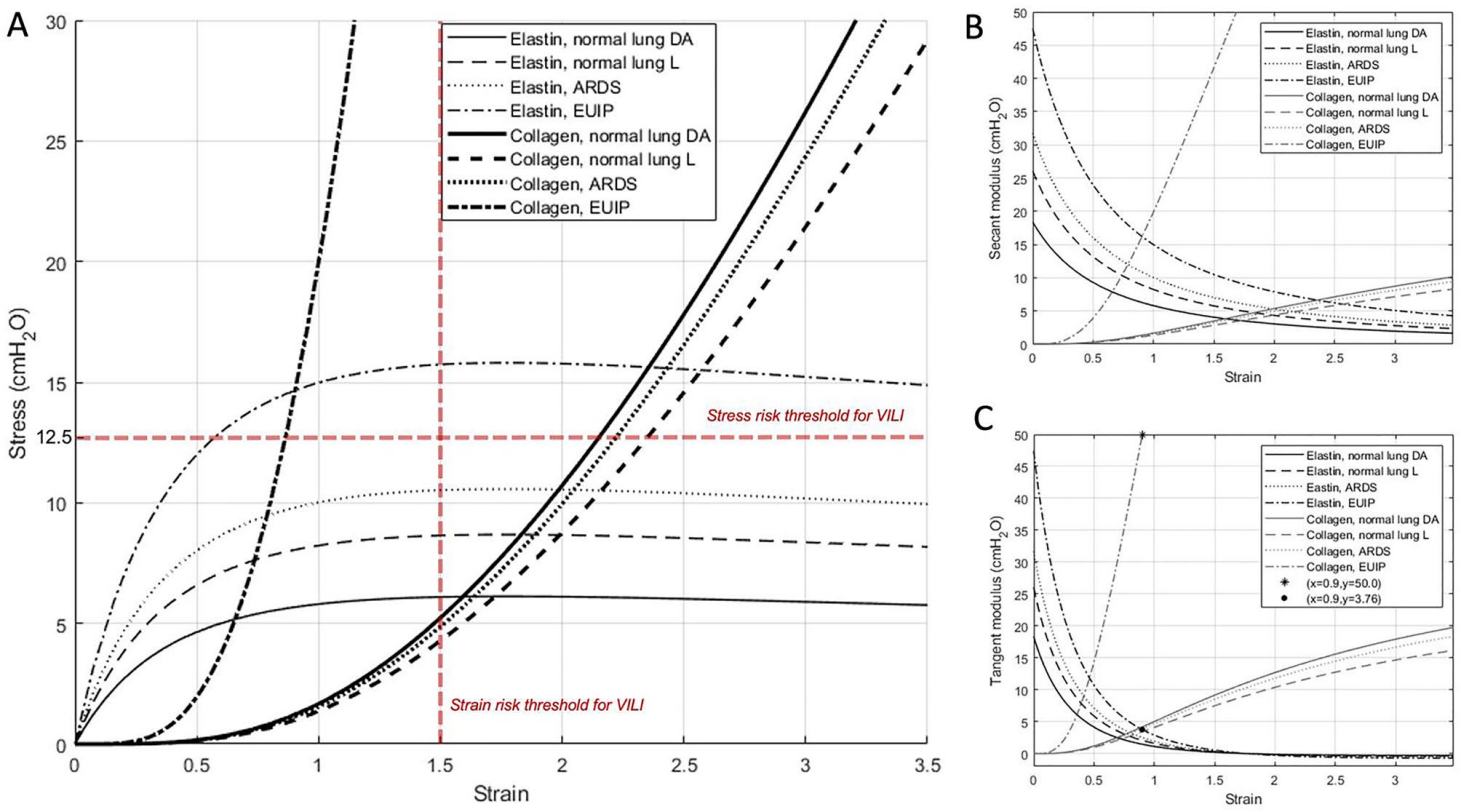
(**Panel A**) Isolated contributions of elastin and ground substance (thin lines) and collagen (thick lines) in the pressure–volume fitting curves. Elastin’s contribution in lung inflation was prevalent at lower strains, while the contribution of collagen was significant at higher strains. With reference to the mechanical model, strain and stress here indicate the relative volume change and the pressure applied inside the composite sphere, respectively. The partitioned analysis of the secant modulus (elastance) and tangent modulus for collagen and elastin of for EUIP, ARDS and healthy patients^14,15^ is shown in (**panels B** and **C**), respectively. For strain value > 0.5 the tangent modulus of elastin showed a steep decrease, and the tangent modulus of collagen showed a marked increase. At strain = 0.9 the modulus of elastin resulted 3.8 cmH_2_O and the modulus of collagen 50.0 cmH_2_O. EUIP, acute exacerbation of interstitial lung disease with usual interstitial pneumonia pattern; ARDS, acute respiratory distress syndrome.

Figure 4, panel A shows the isolated contributions of elastin plus ground substance and collagen in the pressure–volume curves. The contribution of elastin was prevalent at lower strains, while the contribution of collagen was significant at higher strains. The curve for collagen showed an upward shift passing from normal to EUIP lungs. The partitioned analysis of the secant and tangent modulus for collagen and elastin of EUIP patients is shown in panel B and panel C, respectively. For strain value > 0.5 the tangent modulus of elastin showed a steep decrease, and the tangent modulus of collagen showed a marked increase. At strain = 0.9 the two moduli resulted 3.8 cmH_2_O for elastin and 50.0 cmH_2_O for collagen. The piecewise distributions of the radial and hoop stress components, ${\sigma }_{r}$ and ${\sigma }_{\theta }$ respectively, in three deformed (inflated) configurations of the alveolus, corresponding to three different values of the relative volume change $\frac{\Delta V}{V}$ is shown in eFigures 3 and 4, Supplementary for EUIP and ARDS patients respectively.

## Discussion

First and for the first time in literature, we described the mechanical properties of fibrotic lungs by comparing their specific elastance with that of ARDS; based on these data, we proposed a constitutive model for lung deformation during mechanical ventilation, which takes into account the contributions of elastin and collagen – the main components of the ECM. This model was developed with stress/strain data from patients with EUIP and ARDS undergoing MV and fills a theoretical knowledge gap concerning the risk of VILI experienced by patients with EUIP during MV.

### Are data on respiratory mechanics of UIP-lung really needed?

Although the use of MV in patients with AE-ILD and UIP pattern is limited due to unfavorable outcome^3^, the use of invasive respiratory assistance is required in certain cases (bridge to transplantation, ILD of unknown etiology presenting with AE at its onset, overlap with potentially revertible infectious disease)^9^. In this scenario, current guidelines on IPF (the most frequent ILD with UIP pattern) management do not provide any recommendation regarding the use of MV^44^. This may be due to the lack of data on the interplay between ventilatory assistance and the mechanical response of the fibrotic lung with UIP pattern. Selecting a P_es_-guided PEEP strategy seems attractive because P_L_ directly reflects alveolar distending pressure, independent of the pressure required to expand the chest wall. Comparing the effect of such a ventilation strategy on respiratory mechanics of ARDS and EUIP patients, we firstly report evidence of lung elastance derangement for these latter. These data may help in reducing the gap of knowledge on the interaction between MV and lung fibrosis.

### To what extent is the specific elastance different between EUIP and ARDS

The complete unfolding of the collagen fibers at the molecular level, limits the safe maximum lung inflation at total lung capacity (TLC). Studies in patients with early-phase ARDS, found that specific elastance falls within ranges comparable to healthy lungs (about 12 cmH_2_O)^5,8^, which is confirmed by our data. Following the mechanical laws at the basis of VILI, the increased risk of injury starts when crossing the maximal inspiratory capacity. For ARDS lung it corresponds to a stress of 24 cmH_2_O^5^ (the double of specific elastance). As stress results from product of specific elastance and strain, injury can occur for values of specific elastance much lower than the theoretical stress threshold. The specific lung elastance of EUIP resulted significantly higher than that of ARDS, probably reflecting the intrinsic mechanical features of the lung parenchyma which depends on lung structural composition (i.e. ECM). Nevertheless, even if EUIP patients have a specific elastance of nearly 30 cmH_2_O, stress does not have to cross that threshold to be considered harmful. That is to say that in patients with lung fibrosis specific elastance does not result informative on the limit to safe inflation during MV.

### Is there a harmful strain threshold for UIP-lung?

**Figure 5:**
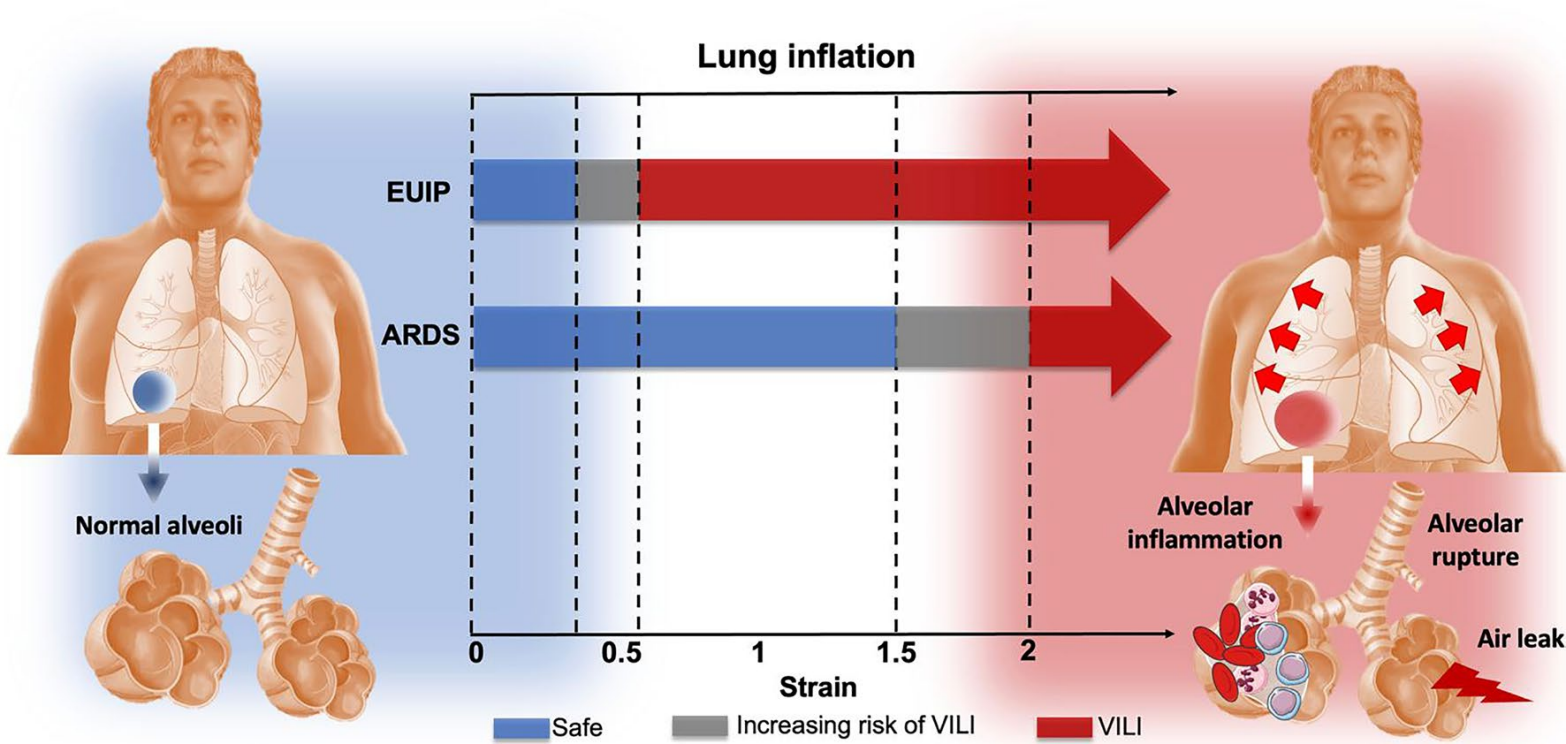
Simplified diagram illustrating the strain threshold predisposing to VILI for EUIP and ARDS. For these latter the risk of VILI starts increasing at 1.5 and becomes significant above 2^5,45^, resulting in lung inflammation and alveolar rupture. Our data show that in patients with UIP-lung the harmful strain threshold starts for strain value of 0.35 and could be reached slightly above 0.5. EUIP, Acute exacerbation of interstitial lung disease with usual interstitial pneumonia pattern; ARDS, Acute respiratory distress syndrome.

Studies conducted on patients with acute lung injury reported that the harmful value of global strain is around 2^5,45^. This is confirmed by our data where ARDS patients showed the highest stress values for strain between 1.5 and 2. Conversely, none of the EUIP reached levels of strain above 1, not even for higher PEEP values. As such, the classical paradigm derived from ARDS in which MV produces clinically relevant VILI for strain ≥ 1^46^ could not fit for the UIP-lung. The global and partitioned analysis of the model fit with EUIP data shows that the stress–strain curve starts rearing up thus deranging its mechanical behavior for values of global strain within 0.35 and 0.55; further, when assessing the contribution of elastin and collagen to lung deformation, our data show that for strain value > 0.5 the tangent moduli start diverging significantly. Thus, we propose it as a threshold risk for VILI development in UIP-lung. Furthermore, we must also consider that in ARDS patients undergoing MV, lethal strain values are rarely applied in clinical practice; indeed, VILI is usually explained through the mechanism of lung parenchyma heterogeneity acting as a “stress riser” at the interface areas between healthy and diseased lung^47,48^. This is even more true for the parenchymal inhomogeneity characterizing the UIP lung, (causing the rise of local stress, see the stress distributions shown in eFigures 3 and 4, Supplementary) where stress raisers take place at the interface of regions characterized by different elastic recoil (also with a “squishy” behavior, which could occur at the microscale^49^). Thus, it is not surprising that the stress–strain curve that we reported for patients with EUIP shows how harmful strain threshold could be reached slightly above 0.5, which is a value commonly applied in clinical practice. Based on the presented data and model, it seems not possible to invasively ventilate patients with EUIP without provoking a certain degree of VILI. This concept is illustrated in a simplified graphic diagram in Fig. 5. However, despite the suggestion offered by our mechanical model, conducting additional research centered on histopathological outcomes in mechanically ventilated ILD patients would aid in determining if lung damage occurs at varying mechanical thresholds. This would ultimately improve patient care while reducing the risk of VILI. For detailed discussion on the mechanical characteristics and implications of the model^22,28^, we refer to the Supplementary.

### Study novelties

Even though alveolar models already exist in the literature ^22,23,39^, our modeling approach is based on two novel aspects. First, we model the alveolus as a spherical shell instead of a spherical membrane as in Jawde et al.^22^ and Shi et al. ^23^. As a consequence, the collagen volume fraction is a model parameter, and it becomes possible to estimate it by comparing the predicted pressure–volume response with the data collected in patients. Next, the choice of the strain energy densities for elastin and collagen (see Eqs. (16, 17) in the Supplementary) has never been proposed before in alveolar shell models, to the knowledge of the authors. The application of the predicted pressure–volume relation (Eq. (18) in the Supplementary) to compare different populations of patients (healthy, ARDS and EUIP) is another element of novelty. Fitting Eq. (18) to the data collected either from the literature (for healthy subjects) or from the retrospective study (for ARDS and EUIP patients) allows to estimate the predicted collagen volume fraction in the different patients’ population. The original result was an increase of the predicted collagen volume fraction in EUIP patients compared with the other cohorts. Biochemical evidence for an increased and progressive deposition of collagen in lungs of patients with pulmonary fibrosis had been widely established^40^. In this line, measures based on atomic force microscopy has demonstrated that EUIP lungs are characterized by a higher elastic modulus compared to healthy lungs^28^. Very recently, an increase in the activity of the protein HIF has been shown to correlate with increase and strengthen of cross-links, between fibers of collagen. This structural alteration stiffens the tissue, triggering cells in the lung to deposit more collagen and starts the process of fibrosis^50^. The increase in the estimate for ${\chi }_{2}$ for EUIP lungs also correlates with the results of studies demonstrating an increase in total collagen type I and III in the composition of the ECM in the early stages of lung fibrosis, with type III collagen predominating in the thickened alveolar septa^51^. An increase of type III collagen in sites of early active fibrosis agrees with observations in other organs and tissues: the proportion of type III collagen in mature rat skin increases from about 10% to 30–40% in acute and chronic inflammatory fibrosis^43^. Our model shows that the role of elastin was predominant in driving deformation at lower strains, whereas the contribution of collagen became significant at higher strains. This is in line with the theoretical findings of the mechanical model proposed by Jawde et al.^22^, and correlates with experimental observations showing that lung parenchyma samples treated with collagenase have a larger drop in stiffness at higher strains, indicating a larger collagen contribution at this stage^35^.

### Study limitations

Our study suffers from several limitations. First, we reported ΔP_L_ values that resulted higher than ΔP_aw_ in the EUIP group at each PEEP level and in the ARDS group at PEEP_TITRATED_. A potential explanation for this might be that the measurement of ΔP_aw_ was performed and provided by the respirator’s software while the measurements of ΔP_L_ were provided by the Optivent™ monitor integrating signals with the pressure transducer included in the esophageal catheter. This could have generated slight discrepancy in the measurements. Further, our analysis has been conducted on just 5 patients with EUIP. However, given that patients with ILD-UIP rarely undergo MV, to our knowledge no data on respiratory mechanics in this subset are available. Secondly, we limited our investigation to patients experiencing AE of disease. Third, we did not provide data on CT images at different PEEP levels nor on biomarkers of lung damage. Additionally, the mathematical model has several simplifying aspects. In this line, the analysis is conducted at the scale of a single alveolus, so regional heterogeneities caused by gravity or intrinsic local tissue variability are neglected. Next, the model focuses on elastic effects on mechanical fields for static inflation, so viscoelasticity is not included in this analysis. Further, the effect of lung surfactant is not considered in the model. Next, we acknowledge that exacerbated ILDs and ARDS differ in terms of outcomes. However, our study population was matched based on baseline gas exchange impairment, which reflects the severity of acute lung injury. The increased mortality observed in MV-UIP patients is consistent with existing literature and underscores the distinct nature of these conditions^9^.

Further possible limitations of the proposed study arise from the assumptions used for data extraction of healthy lungs from the work of D’Angelo et al.^14^ and Levy et al.^15^. In addition, patients studied by D’Angelo et al.^14^ seem to be younger. Because data from normal lungs are relevant for the present study, the collection of these data needs to be improved and will be the subject of further studies. Despite these limitations, we believe that our results could pave the way for further research in the clinical management of patients with EUIP in need of MV. Indeed, our data seem to suggest that even when the principles of protective MV are applied, patients with EUIP still are at high risk of VILI. This raises the issue on how to manage EUIP patients with no ceiling of escalation to MV (i.e. bridge to transplantation). In this scenario whether alternative strategies (i.e. ultra-protective ventilation, extra-corporeal membrane oxygenation) may limit the application of harmful airways pressure to support vital function should be matter of further research.

## Conclusion

The stress/strain curve of patients with fibrotic lung and UIP pattern during exacerbation of disease explains why these patients may experience harmful values of lung stress during invasive ventilatory assistance even when the principles of lung protective MV developed for ARDS are applied. According to our novel mathematical model, we suggest that the peculiar model of deformation in patients with fibrotic lung and UIP might be due to the different contribution of the main ECM components. Our model fills a theoretical research gap and encourage research on a tailored management of UIP-lung during exacerbation.

### Supplementary Information

Supplementary Information 1.

Supplementary Information 2.

Supplementary Information 3.

Supplementary Information 4.

## Data Availability

The datasets used and/or analysed during the current study are available from the corresponding author on reasonable request.

## Abbreviations

ILD: Interstitial lung disease
AE: Acute exacerbation
UIP: Usual interstitial pneumonia
ARDS: Acute respiratory distress syndrome
AHRF: Acute hypoxic respiratory failure
bpm: Breaths per minute
MV: Invasive mechanical ventilation
ETI: Endotracheal intubation
PEEP: Positive end-expiratory pressure
PBW: Predicted body weight
PSV: Pressure support
APACHE II: Acute physiology and chronic health evaluation II
SAPS II: Simplified acute physiology score
IPF: Idiopathic pulmonary fibrosis
RICU: Respiratory intensive care unit
ICU: Intensive care unit
P_es_: Esophageal pressure
P_es,EI_: End-inspiratory esophageal pressure
P_es,EE_: End-expiratory esophageal pressure
P_L_: Transpulmonary pressure
∆P_L_: Transpulmonary driving pressure
P_L,EI_: End-inspiratory transpulmonary pressure
P_L,EE_: End-expiratory transpulmonary pressure
P_plat_: End-inspiratory plateau pressure
∆P_aw_: Driving pressure
E_tot_: Respiratory system elastance
E_cw_: Chest wall elastance
E_L_: Lung elastance
FRC: Functional residual capacity
IQR: Interquartile range
