# Supplementary material for: Stress–strain curve and elastic behavior of the fibrotic lung with usual interstitial pneumonia pattern during protective mechanical ventilation
Source: Sci Rep. 2024 Jun 7;14:13158. doi: 10.1038/s41598-024-63670-z (PMC11161630; doi:10.1038/s41598-024-63670-z)
Supplement: Supplementary file 1 — Supplementary Information 1. [file 41598_2024_63670_MOESM1_ESM.docx]

**Supplement 1**

*Mechanics variables definition*

End-inspiratory transpulmonary pressure (P_L,EI_) was computed according to the formula

P_L,EI_ = P_plat_ – P_es,EI_

where P_plat_ is end-inspiratory plateau pressure and P_es, EI_ is end-inspiratory esophageal pressure.

Lung stress was defined as P_L,EI_.

The EELV measure was based on the nitrogen washout/washin technique through dedicated software (FRC Inview, GE Healthcare). The principle of this technique is as follows: The volume of lung gas includes a volume of nitrogen (V _(1)_ N_2_) that is determined by alveolar fraction of nitrogen (F_A_N_2(1)_) and by the EELV:

V_(1)_N_2_ = F_A_N_2(1)_ X EELV

The alveolar fraction of nitrogen can change by changing the administered FiO2 (F_A_N_2(2)_), resulting in a new volume of nitrogen (V_(2)_N_2_) in the lung after a period of balance.

V_(2)_N_2_ = F_A_N_2(2)_ X EELV

Assuming that after the change of FiO2 the EELV does not change, until a new balance of the alveolar gas is reached, the following equation can be written:

V_(1)_N_2_ – V_(2)_N_2_ = (F_A_N_2(2)_ –FAN_2(1)_) X EELV

As the changes in F_A_N_2_are specular to the changes in FiO_2_ the EELV can be calculated as:

EELV = ΔN_2_(ml)/ΔFiO_2_

where ΔN_2_ is equal to the exhaled nitrogen after the change in FiO_2_ once equilibrium has been reached (20 breaths). The algorithm used by the Engstrom Carestation (FRC Inview, GE Healthcare) employs tidal concentration of oxygen and carbon dioxide to obtain an estimate of nitrogen concentration in expired and inspired air. For more details, see reference 10 and 20.

Global lung strain was defined according to the formula

Global strain = ΔV/FRC

where ΔV was computed as follows: EELV – FRC + Vt^6,10^ and FRC is functional residual capacity.

Specific elastance was computed according to the formula

Specific elastance = P_L,EI_ /(ΔV/FRC)

**eFigure 1**

**
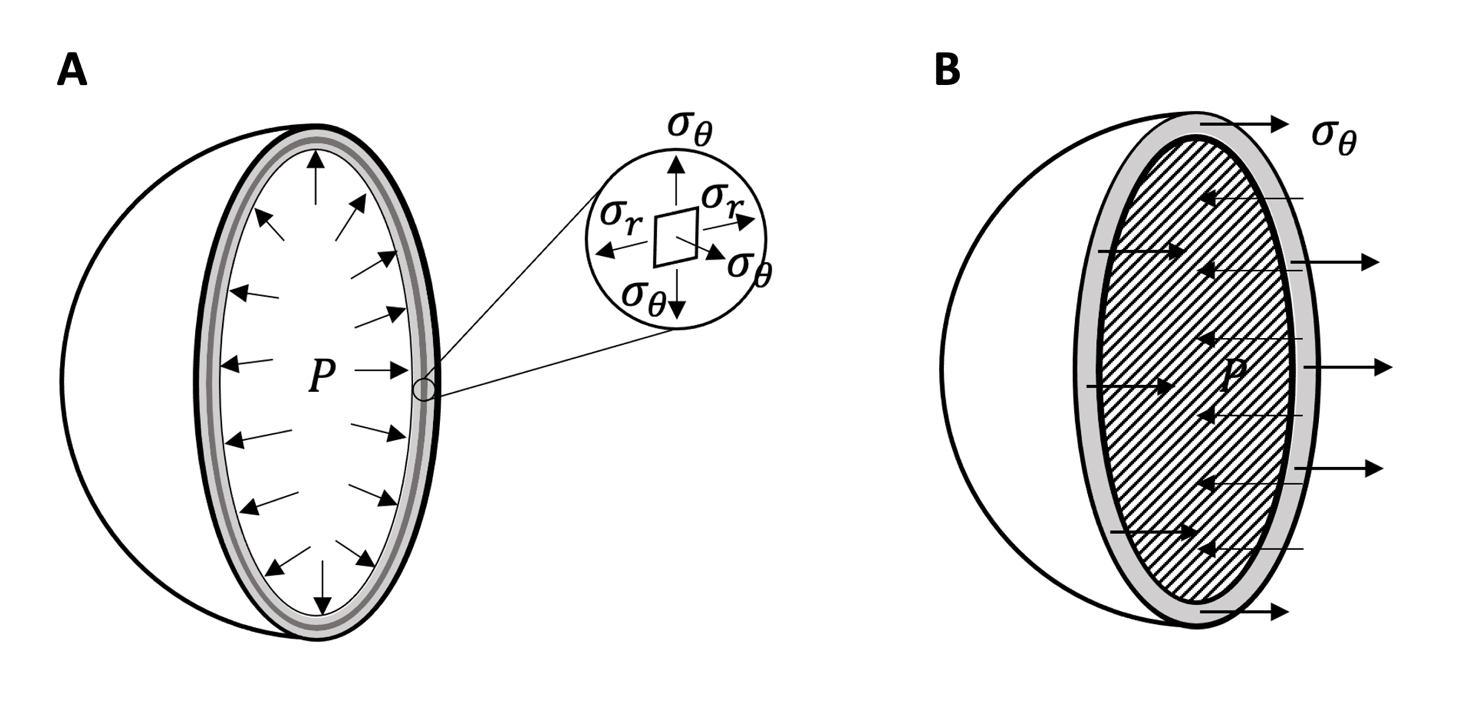
**

**eFigure 1. Panel A.** geometry of the three-layered shell modeling an alveolus. Dark gray indicates a layer of collagen interposed between two layers composed of elastin and ground material, colored in light gray. The shell is inflated by an inner pressure $P.$ The inset shows the stress components acting at each point of the shell material: the radial stress component, $\sigma_{r}$, is in the radial direction, and the circumferential (or hoop) stress components, $\sigma_{\theta}$ and $\sigma_{\varphi}$ (here $\sigma_{\varphi}=\sigma_{\theta},$ due to axial symmetry), are in the tangential direction of the meridians and the parallels of the sphere. **Panel B.** For a homogeneous shell, force equilibrium requires the resultant of $\sigma_{\theta},$ acting on the small area depicted in gray, to be much larger than the resultant of the pressure $P$ acting on the dashed larger area.

*Equilibrium of a pressurized composite shell*

Consider a spherical shell made of $n$ layers composed of incompressible isotropic hyperelastic materials. Let $X=X\left( R,\Theta,\Phi\right)$ denote the position of a material particle in the undeformed conﬁguration, where the geometry is defined as

$R_{i}\leq R\leq R_{e},$ $0\leq\Theta\leq\pi,$ $0\leq\Phi\leq2\pi,$ (1)

with $R_{i}$ and $R_{e}$ the inner and outer radii of the sphere, respectively. The sphere is divided into $n$ layers, being the $j$-th layer comprised between the radii $R_{j}$ and $R_{j+1}$, $j=1, 2,\ldots n,$ with $R_{1}=R_{i}$ and $R_{n+1}=R_{e}$. Let $h_{j}:=R_{j+1}-R_{j}$ denote the thickness of the $j$-th layer and $\varepsilon_{n+1}:=\frac{h}{R_{i}}$ to denote the (small) ratio of total thickness $h$ to internal radius $R_{i}$.

The multilayered shell is subject to a uniform internal pressure $P$. Let $x=x\left( r,\theta,\varphi\right)$denote the position of a material particle in the deformed conﬁguration and let spherical symmetry be retained during inflation, so that a finite deformation of the shell is given by

$r=f\left( R \right), \theta=\Theta, \varphi=\Phi.$ (2)

The deformed geometry is described by

$r_{1}\leq r\leq r_{n+1},$ $0\leq\theta\leq\pi,$ $0\leq\varphi\leq2\pi.$ (3)

with $r_{1}$ and $r_{n+1}$the inner and outer radii of the deformed conﬁguration, respectively.

The principal stretches are $\lambda_{r}=\frac{dr}{dR}$ in the radial direction and $\lambda_{\theta}=\lambda_{\varphi}=\frac{r}{R}=:\lambda$ in the circumferential directions, respectively. Incompressibility requires that $\lambda_{r}=\lambda^{-2}$ and radial deformation can be calculated as

$f\left( R \right)=\left( R^{3}+r_{1}^{3}-R_{1}^{3} \right)^{\frac{1}{3}}.$ (4)

By continuity, the latter equation holds for $R_{1}\leq R\leq R_{n+1}.$ We also introduce the following notation: $r_{j}:=f\left( R_{j} \right)$, and $\lambda_{j}:=\frac{r_{j}}{R_{j}}$, $j=1, 2,\ldots n,$ being $\lambda_{j}$ the stretches at the inner surfaces between the layers. Using Equation (4), these stretches can be related to the relative volume change $\frac{\Delta V}{V}$ inside the sphere as follows:

$\lambda_{j}=\left( 1+\frac{1}{{(1+\varepsilon_{j})}^{3}}\frac{\Delta V}{V} \right)^{1/3},$ (5)

with $\varepsilon_{j}:=(R_{j}-R_{1})/R_{1}$, $j=1, 2, \ldots n+1.$ Substituting the relation $R_{j}=R_{1}+\sum_{k=1}^{j-1} h_{k}$ into the expression of $\varepsilon_{j}$, we find

$\varepsilon_{j}:=\frac{\sum_{k=1}^{j-1} h_{k}}{R_{1}}=\varepsilon_{n+1}\sum_{k=1}^{j-1} \chi_{k}, j=2,\ldots n+1,$ (6)

with $\chi_{k}:=h_{k}/h$ the fraction of wall thickness of the $k-$th layer. For a very thin shell, the thickness fraction can be approximated by the volume fraction of the $k-$th layer up to a term $O(\varepsilon_{n+1})$. By definition $\varepsilon_{1}=0,$ thus the stretch at the inner surface is simply given by

$\lambda_{1}=\left( 1+\frac{\Delta V}{V} \right)^{1/3}.$ (7)

Note that, in view of Eqn. (7), we have $\lambda_{j}>\lambda_{j+1},$ $j =1, 2\ldots n+1.$

The behavior of the material composing the $j$-th layer be described by the strain energy density $W^{\left( j \right)}\left( \lambda_{r},\lambda_{\theta},\lambda_{\varphi} \right)$, function of $\lambda_{r},\lambda_{\theta},\lambda_{\varphi}$ via the strain invariants. The Cauchy principal stress components in the $j$-th layer are given by

$\sigma_{r}^{\left( j \right)}=\lambda_{r}\frac{\partial W^{\left( j \right)}}{\partial\lambda_{r}}-p^{\left( j \right)}, \sigma_{\theta}^{\left( j \right)}=\sigma_{\varphi}^{\left( j \right)}=\lambda_{\theta}\frac{\partial W^{\left( j \right)}}{\partial\lambda_{\theta}}-p^{\left( j \right)}, j =1, 2\ldots n+1,$ (8)

where $p^{\left( j \right)}, j=1, 2\ldots n+1,$ are arbitrary hydrostatic pressures. Axial symmetry implies that the only nontrivial equilibrium equations are

$\frac{d\sigma_{r}^{\left( j \right)}}{dr}+2\frac{{(\sigma}_{r}^{\left( j \right)}-\sigma_{\theta}^{\left( j \right)})}{r}=0, j =1, 2, \ldots n+1.$ (9)

After introducing the auxiliary functions $w^{\left( j \right)}\left( \lambda\right):=W^{\left( j \right)}\left( \lambda^{-2},\lambda,\lambda\right), j=1, 2, \ldots n+1$, it can be shown that Equations (6) are equivalent to

$\frac{d\sigma_{r}^{\left( j \right)}}{d\lambda}=-\frac{\dot{w}^{\left( j \right)}}{\lambda^{3}-1}, j=1, 2, \ldots n+1,$ (10)

where the dot indicates differentiation with respect to $\lambda ADDIN ZOTERO\_TEMP$^20^. After integrating and imposing the boundary conditions $\sigma_{r}^{\left( 1 \right)}\left( \lambda_{1} \right)=-P$ and $\sigma_{r}^{\left( N \right)}\left( \lambda_{N+1} \right)=0$, we obtain that

$\sigma_{r}^{\left( 1 \right)}\left( \lambda\right)=\int_{\lambda}^{\lambda_{1}} \frac{\dot{w}^{\left( 1 \right)}\left( l \right)}{l^{3}-1} dl-P, \lambda_{2}\leq\lambda\leq\lambda_{1},$ (11)

$\sigma_{r}^{\left( j \right)}\left( \lambda\right)=\int_{\lambda}^{\lambda_{j}} \frac{\dot{w}^{\left( j \right)}\left( l \right)}{l^{3}-1} dl+\sum_{k=1}^{j-1} \int_{\lambda_{k+1}}^{\lambda_{k}} \frac{\dot{w}^{\left( k \right)}\left( l \right)}{l^{3}-1} dl-P, \lambda_{j+1}\leq\lambda\leq\lambda_{j}, j=2, \ldots n+1,$ (12)

$P=\sum_{j=1}^{n} \int_{\lambda_{j+1}}^{\lambda_{j}} \frac{\dot{w}^{\left( j \right)}\left( l \right)}{l^{3}-1} dl.$ (13)

Equation (13) gives the internal pressure as a function of the stretches $\lambda_{j}$. Finally, given $\sigma_{r}^{\left( j \right)}\left( \lambda\right)$ from Equations (8,9), the circumferential stress can be calculated using Equation. (6) as follows:

$\sigma_{\theta}^{\left( j \right)}\left( \lambda\right)=\frac{1}{2}{\lambda\dot{w}^{\left( j \right)}\left( \lambda\right)+\sigma}_{r}^{\left( j \right)}\left( \lambda\right)$, $\lambda_{j+1}\leq\lambda\leq\lambda_{j},$ $j=1, 2, \ldots n+1.$ (14)

As discussed in ^21^, in normal test conditions alveoli can be considered thin shells with a thickness to radius ratio $\varepsilon_{n+1}$close to 0.05. Thus, each $\varepsilon_{j}$ in Equation (11) can be assumed to be a small parameter. Substituting Equations (11) and (12) into Equation (10) and expanding to first order with respect to $\varepsilon_{n+1}$ leads to the following approximation

$P=\varepsilon_{n+1}\sum_{j=1}^{n} \chi_{j}\left( \frac{\dot{w}^{\left( j \right)}(\lambda_{1})}{{\lambda_{1}}^{2}} \right),$ (15)

with $\lambda_{1}$ given by Equation (13). This equation is the formula for inflation pressure for a multilayered balloon. The case of homogenous balloon is considered for example in ^22^.

*Constitutive modelling of collagen and elastin*

Collagen fibers display a high nonlinear behavior and stiffening at large strains, while elastin and ground substance are softer. A simple choice for their strain energy densities is

$W^{\left( 1 \right)}\left( \lambda_{r},\lambda_{\theta},\lambda_{\varphi} \right)=c_{1}\left( \lambda_{r}^{2}+\lambda_{\theta}^{2}+\lambda_{\varphi}^{2}-3 \right),$ (16)

$W^{\left( 2 \right)}\left( \lambda_{r},\lambda_{\theta},\lambda_{\varphi} \right)=c_{2}\left( \lambda_{r}^{2}+\lambda_{\theta}^{2}+\lambda_{\varphi}^{2}-3 \right)^{3},$ (17)

with $c_{1}, c_{2}$ positive material constants. This choice agrees with the constitutive model of lung parenchyma experimentally determined via uniaxial tension tests on living precision-cut rat lung slices^23^. The main difference between Equations (16), (17) and the constitutive model proposed by Rausch and coworkers is that here we assume the materials to be incompressible, while they account for an energy term to control the volumetric change. We also note that the stress-strain response in traction of the constitutive model (17) can be interpreted as a phenomenological description of the toe region followed by gradual, nonlinear stiffening of curly collagen fibers, due to progressive recruitment of fibers characterized by different amounts of slackness^24^. For multiscale modeling of collagen and proteins with unfolding domains the reader is referred to the literature ^21,25–27^. It is worth mentioning that while the material constant $c_{1}$ is proportional to the initial elasticity modulus of elastin in traction test, the material constant $c_{2}$ can not be thought to represent such a quantity for collagen. Indeed, the stress-strain relation in traction corresponding to the energy given by Equation (17) presents a vanishing slope at the origin, corresponding to zero initial elasticity modulus. So, an increase of $c_{2}$ can be more appropriately interpreted as an increase of collagen stiffening occurring at larger strains.

*Pressure-volume relation*

Even though the present model allows for the description of multiple layers of collagen alternated to layers composed of elastin and ground material, for simplicity we assume the alveolus made of only three layers, the two external ones composed of elastin and ground material, and one in between composed of collagen.

We take$\chi_{2}$ to denote the thickness fraction of collagen, and $1-\chi_{2}$ the remaining fraction of elastin and ground substance. With this notation, the pressure-volume for the shell model can be obtained by substituting Equations (16,17) into Equation (13). We find that

$P=\mathcal{-g}_{1}\left( \lambda_{2} \right)+\mathcal{g}_{1}\left( \lambda_{1} \right)-\mathcal{g}_{2}\left( \lambda_{3} \right)+\mathcal{g}_{2}\left( \lambda_{2} \right)-\mathcal{g}_{1}\left( \lambda_{4} \right)+\mathcal{g}_{1}\left( \lambda_{3} \right),$ (18)

with

$\mathcal{g}_{1}\left( z \right):= \int\frac{\dot{w}^{\left( 1 \right)}\left( l \right)}{l^{3}-1} dl=-\frac{c_{1}}{z^{4}}-\frac{4c_{1}}{z}$, (19)

$\mathcal{g}_{2}\left( z \right):= \int\frac{\dot{w}^{\left( 2 \right)}\left( l \right)}{l^{3}-1} dl=3c_{2}(-\frac{1}{3z^{12}}-\frac{4}{9z^{9}}+\frac{3}{z^{8}}-\frac{8}{3z^{6}}+\frac{24}{5z^{5}}-\frac{9}{z^{4}}-\frac{16}{3z^{3}}+\frac{24}{z^{2}}-\frac{36}{z}-48z+\frac{16z^{3}}{3}+16\ln z)$.

(20)

In Equation (18), the stretches $\lambda_{j}, j=1,2,3,4,$ defined by Equation (5), can be more conveniently rewritten in terms of $\chi_{2}$ and $\frac{\Delta V}{V}$as follows:

$\lambda_{2}=\left( 1+\frac{1}{\left( 1+\varepsilon_{n+1}{(1-\chi}_{2})/2 \right)^{3}}\frac{\Delta V}{V} \right)^{\frac{1}{3}},$ (21)

$\lambda_{3}=\left( 1+\frac{1}{\left( 1+\varepsilon_{n+1}{(1+\chi}_{2})/2 \right)^{3}}\frac{\Delta V}{V} \right)^{\frac{1}{3}},$ (22)

$\lambda_{4}=\left( 1+\frac{1}{\left( 1+\varepsilon_{n+1} \right)^{3}}\frac{\Delta V}{V} \right)^{\frac{1}{3}}.$ (23)

Notably, the isolated contributions of elastin (and ground substance) and collagen in Equation (18) are given by the terms containing the functions $\mathcal{g}_{1}$ and $\mathcal{g}_{2}$, respectively.

*Radial and hoop stress distributions*

For the three layers configuration constituted of a layer of collagen interposed between two outermost layers of elastin and ground material, the piecewise distribution of radial stress along the thickness of the deformed shell can be obtained by substituting Equations (16,17) into (11,12) to get the following relation:

$\left\{ \begin{matrix} \sigma_{r}^{\left( 1 \right)}\left( \frac{r}{r_{1}} \right)=-P+\mathcal{g}_{1}\left( \mathcal{l}\left( \frac{r}{r_{1}} \right) \right)-\mathcal{g}_{1}\left( \lambda_{1} \right), \zeta_{1}\leq\frac{r}{r_{1}}\leq\zeta_{2}, \\ \sigma_{r}^{\left( 2 \right)}\left( \frac{r}{r_{1}} \right)=-P+\mathcal{g}_{1}\left( \lambda_{2} \right)-\mathcal{g}_{1}\left( \lambda_{1} \right)+\mathcal{g}_{2}\left( \mathcal{l}\left( \frac{r}{r_{1}} \right) \right)-\mathcal{g}_{2}\left( \lambda_{2} \right), \zeta_{2}<\frac{r}{r_{1}}\leq\zeta_{3}, \\ \sigma_{r}^{\left( 3 \right)}\left( \frac{r}{r_{1}} \right)=-P+\mathcal{g}_{1}\left( \lambda_{2} \right)-\mathcal{g}_{1}\left( \lambda_{1} \right)+\mathcal{g}_{2}\left( \lambda_{3} \right)-\mathcal{g}_{2}\left( \lambda_{2} \right)+\mathcal{g}_{1}\left( \mathcal{l}\left( \frac{r}{r_{1}} \right) \right)-\mathcal{g}_{1}\left( \lambda_{3} \right), \zeta_{3}<\frac{r}{r_{1}}\leq\zeta_{4}, \end{matrix} \right.$ (24)

with

$\mathcal{l}\left( z \right)=\left( \frac{z^{3}}{z^{3}-\frac{\frac{\Delta V}{V}}{1+\frac{\Delta V}{V}}} \right)^{\frac{1}{3}},$ (25)

and $\zeta_{j}:=\frac{r_{j}}{r_{1}}, j=1,2,3,4,$ the ratios between the deformed radius $r_{j}$ at the surfaces between the layers and the inner deformed radius $r_{1}.$ Thus $\zeta_{1}=1,$ while $\zeta_{j}, j=2,3,4$ take the following form in terms of $\chi_{2}$ and $\frac{\Delta V}{V}:$

$\zeta_{1}=\left( \frac{\frac{\Delta V}{V}}{1+\frac{\Delta V}{V}}+\frac{\left( 1+\varepsilon_{n+1}{(1-\chi}_{2})/2 \right)^{3}}{1+\frac{\Delta V}{V}} \right)^{\frac{1}{3}},$ (26)

$\zeta_{2}=\left( \frac{\frac{\Delta V}{V}}{1+\frac{\Delta V}{V}}+\frac{\left( 1+\varepsilon_{n+1}{(1+\chi}_{2})/2 \right)^{3}}{1+\frac{\Delta V}{V}} \right)^{\frac{1}{3}},$ (27)

$\zeta_{3}=\left( \frac{\frac{\Delta V}{V}}{1+\frac{\Delta V}{V}}+\frac{\left( 1+\varepsilon_{n+1} \right)^{3}}{1+\frac{\Delta V}{V}} \right)^{\frac{1}{3}}.$ (28)

From Equation (24), using Equation (14), the piecewise distribution of hoop stress along the thickness of the deformed shell can be shown to take the following expression:

$\left\{ \begin{matrix} \sigma_{\theta}^{\left( 1 \right)}\left( \frac{r}{r_{1}} \right)=\frac{1}{2}\mathcal{l}\left( \frac{r}{r_{1}} \right) \dot{w}^{\left( 1 \right)}\left( \mathcal{l}\left( \frac{r}{r_{1}} \right) \right)+\sigma_{r}^{\left( 1 \right)}\left( \frac{r}{r_{1}} \right), \zeta_{1}\leq\frac{r}{r_{1}}\leq\zeta_{2}, \\ \sigma_{\theta}^{\left( 2 \right)}\left( \frac{r}{r_{1}} \right)=\frac{1}{2}\mathcal{l}\left( \frac{r}{r_{1}} \right) \dot{w}^{\left( 2 \right)}\left( \mathcal{l}\left( \frac{r}{r_{1}} \right) \right)+\sigma_{r}^{\left( 2 \right)}\left( \frac{r}{r_{1}} \right), \zeta_{2}<\frac{r}{r_{1}}\leq\zeta_{3}, \\ \sigma_{\theta}^{\left( 3 \right)}\left( \frac{r}{r_{1}} \right)=\frac{1}{2}\mathcal{l}\left( \frac{r}{r_{1}} \right) \dot{w}^{\left( 1 \right)}\left( \mathcal{l}\left( \frac{r}{r_{1}} \right) \right)+\sigma_{r}^{\left( 3 \right)}\left( \frac{r}{r_{1}} \right), \zeta_{3}<\frac{r}{r_{1}}\leq\zeta_{4}. \end{matrix} \right.$ (29)
