# Supplementary material for: Stress–strain curve and elastic behavior of the fibrotic lung with usual interstitial pneumonia pattern during protective mechanical ventilation
Source: Sci Rep. 2024 Jun 7;14:13158. doi: 10.1038/s41598-024-63670-z (PMC11161630; doi:10.1038/s41598-024-63670-z)
Supplement: Supplementary file 2 — Supplementary Information 2. [file 41598_2024_63670_MOESM2_ESM.docx]

**Supplement 2**

**eTable 1.**

| Parameter | AE-ILD-UIP  (n=5) | ARDS  (n=5) | p value |
| --- | --- | --- | --- |
| **Age, years** | 62 (56 – 68) | 64 (53 – 70) | 0.9 |
| **Male, n** | 8 (80) | 7 (70) | 0.9 |
| **BMI, kg/m^2^** | 22.3 (20.8 – 25) | 22.4 (20.8 – 23.8) | 0.8 |
| **Charlson index, score** | 4 (3 – 5) | 4 (3 – 5) | 0.9 |
| **APACHE, score** | 13 (11.5 – 14) | 14 (12.5 – 14) | 0.7 |
| **SAPS II, score** | 27 (24.5 – 32.5) | 31 (24 – 32) | 0.6 |
| **†PaO_2_/FiO_2_, mmHg** | 90 (77 – 115) | 85 (73 – 111) | 0.8 |
| **RICU/ICU survival, n** | 0 (0) | 3 (60) | 0.01 |

**eTable 1.** General and clinical characteristics in the study groups on admission. Data are presented as number (n) and percentage for dichotomous values or median and IQR for continuous values and number and percentage.

† The values of APACHE score and PaO_2_/FiO_2_ ratio used for matching these groups were those measured at the time of RICU or ICU admission

*AE-ILD-UIP, acute exacerbation of interstitial lung disease with usual interstitial pneumonia pattern; ARDS, acute respiratory distress syndrome; BMI, body mass index; APACHE II, Acute Physiology and Chronic Health Evaluation II; SAPS II; Simplified Acute Physiology Score; RICU, respiratory intensive care unit; ICU, intensive care unit; IQR, interquartile range*

**eFigure 1**


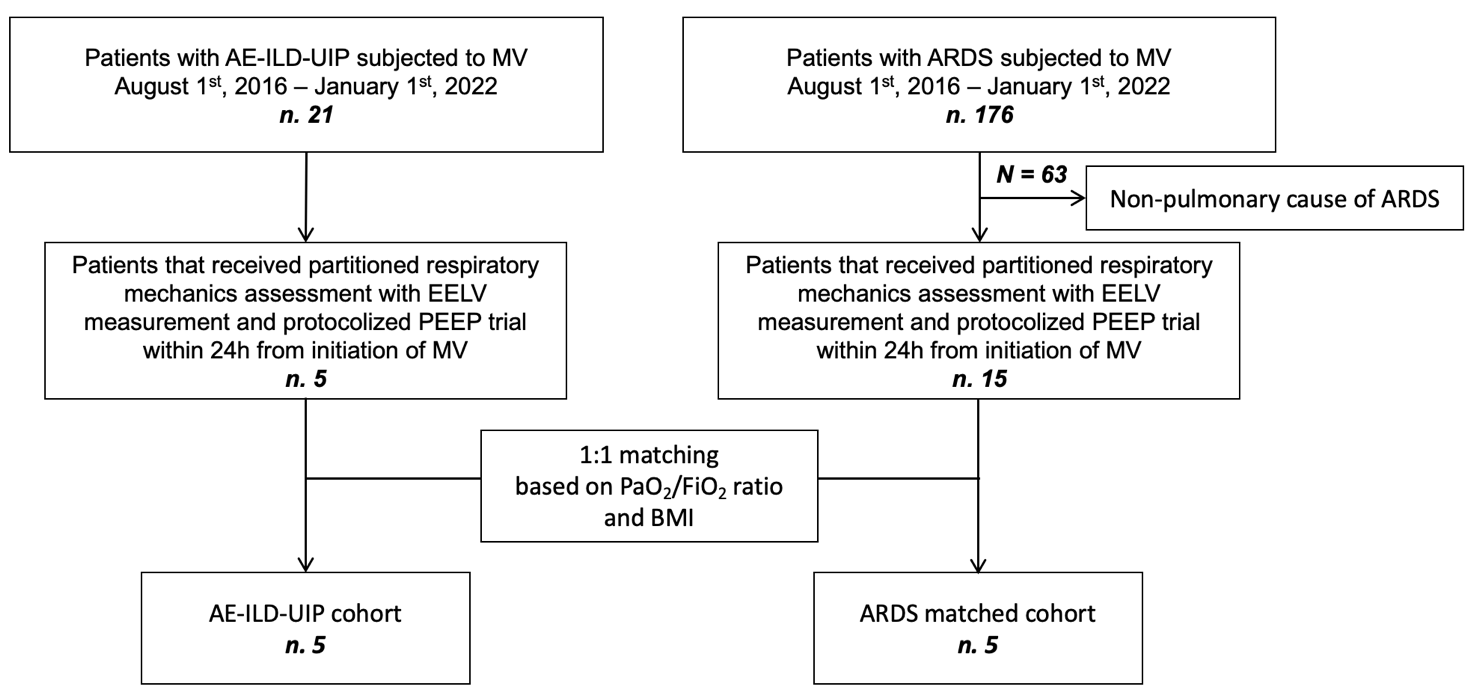
 **eFigure 1.** Study algorithm.

*AE-ILD-UIP, acute exacerbation of interstitial lung disease with usual interstitial pneumonia pattern; ARDS, acute respiratory distress syndrome; RICU, Respiratory Intensive Care Unit; ICU, Intensive Care Unit; MV, mechanical ventilation; PEEP, positive end-expiratory pressure; EELV end-expiratory lung volume; BMI, body mass index*
