# Supplementary material for: Stress–strain curve and elastic behavior of the fibrotic lung with usual interstitial pneumonia pattern during protective mechanical ventilation
Source: Sci Rep. 2024 Jun 7;14:13158. doi: 10.1038/s41598-024-63670-z (PMC11161630; doi:10.1038/s41598-024-63670-z)
Supplement: Supplementary file 3 — Supplementary Information 3. [file 41598_2024_63670_MOESM3_ESM.docx]

**Supplement 3**

*Mechanical considerations*

The lower values of $c_{1}$ and $c_{2}$ in **Table 2**, estimated for healthy human lungs, are comparable with those reported by Rausch and coworkers, $c_{1}$= 4.1 kPa and $c_{2}$= 20.7 kPa, obtained by fitting experimental data of uniaxial tension tests on living precision-cut rat lung slices^23^. The values estimated for $\chi_{2}$ in **Table 2** for normal and ARDS lungs agree with the findings of Mercer and Crapo, who report a mean of 7% for the volume fraction of collagen in healthy human lungs far from the alveolar duct^40^. For AE-ILD-UIP patients, **Table 2** shows an increase in the elastic parameters $c_{1}$ and $c_{2}$, as well as an increase in the collagen fraction $\chi_{2}.$ Measures based on atomic force microscopy has demonstrated that UIP lungs are characterized by a higher elastic modulus compared to healthy lungs^31^. Very recently, an increase in the activity of the protein HIF has been shown to correlate with increase and strengthen of cross- links, between fibers of collagen. This structural alteration stiffens the tissue, triggering cells in the lung to deposit more collagen and start the process of fibrosis^41^. The increase in the estimate for $\chi_{2}$ for AE-ILD-UIP lungs also correlates with the results of studies demonstrating an increase in total collagen type I and III in the composition of the ECM in the early stages of lung ﬁbrosis, with type III collagen predominating in the thickened alveolar septa^42^. Biochemical evidence for an increased and progressive deposition of collagen in lungs of patients with pulmonary fibrosis had been also established in ^33^. An increase of type III collagen in sites of early active fibrosis agrees with observations in other organs and tissues: the proportion of type III collagen in mature rat skin increases from about 10% to 30-40% in acute and chronic inflammatory fibrosis ^43^.

The mechanical contributions of elastin and collagen depicted in **Figure 4** is in line with the theoretical findings of the mechanical model proposed by Jawde at al.^21^, and correlates with experimental observations showing that lung parenchyma samples treated with collagenase have a larger drop in stiffness at higher strains, indicating a larger collagen contribution at this stage^44^.

**eFigure 3** and **eFigure 4** show the piecewise distributions of the radial and hoop stress components, $\sigma_{r}$ and $\sigma_{\theta}$ respectively, in three deformed (inflated) configurations of the alveolus, corresponding to three different values of the relative volume change $\frac{\Delta V}{V}$, with V=FRC. The plots have been obtained by inserting the values of the material parameters listed in Table 3 into Equations (24-29). For normal lungs (DA and L data) and ARDS lungs, the following values of the relative volume change have been considered: $\frac{\Delta V}{V}=0.25, 0.75$ and $1.5$. For AE-ILD-UIP lungs, characterized by a steeper pressure-volume response, the distributions have been plotted for $\frac{\Delta V}{V}=0.25, 0.75$ and $0.85.$ In **eFigure 3** and **eFigure 4**, the stress components $\sigma_{r}$ and $\sigma_{\theta}$ have been represented versus the normalized radial position in the deformed configuration, $\frac{r}{r_{1}},$ with $r$ the radial position and $r_{1}$ the (deformed) radius of the internal shell surface. The normalized radial position ranges from 1 to $\zeta_{4},$ with $r_{4}=\zeta_{4} r_{1}$ the (deformed) radius of the external shell surface. As $\frac{\Delta V}{V}$ increases the shell gets thinner, and correspondingly $\zeta_{4}$ and the domain on the horizontal axis shrink.

Equilibrium at the interfaces between the layers implies the stress component $\sigma_{r}$ to be a continuous function of $\frac{r}{r_{1}}$. Equilibrium at the boundary implies $\sigma_{r}$ to equal $-P$ at the inner surface and to vanish at the outer surface.

The discontinuity of the stress component $\sigma_{\theta}$ along the sphere thickness, i.e., with respect to $\frac{r}{r_{1}},$ is due to the inhomogeneity of the material. The plots on the bottom of **eFigure 3** and **eFigure 4** show that $\sigma_{\theta}$ takes values up to two orders of magnitude greater than $\sigma_{r}.$ For a homogeneous alveolus, this effect would be due to equilibrium, as schematically represented on the right-hand side of Figure 1, where the case of a homogeneous sphere is shown. Considering for simplicity the case of a very thin shell (a balloon) of radius $R$ and thickness $h$, force equilibrium requires ${2\pi\mathrm{Rh}\sigma}_{\theta}=\pi R^{2}P,$ implying $\sigma_{\theta}=\frac{\mathrm{RP}}{2h}.$For a ratio $h/R$ of the order of 0.05 (like in normal lungs, see reference ^21^), $\sigma_{\theta}$ turns out to be one order of magnitude greater than the pressure $P.$ The internal collagen layer, undergoing increasing stiffening for large strains, withstands an even larger stress, that can reach over two orders of magnitude the value of the internal pressure, as indicated by the distribution of hoop stress for the largest values $\frac{\Delta V}{V}$in the plots on the bottom of **eFigure 3** and **eFigure 4.**

**eFigure 3**


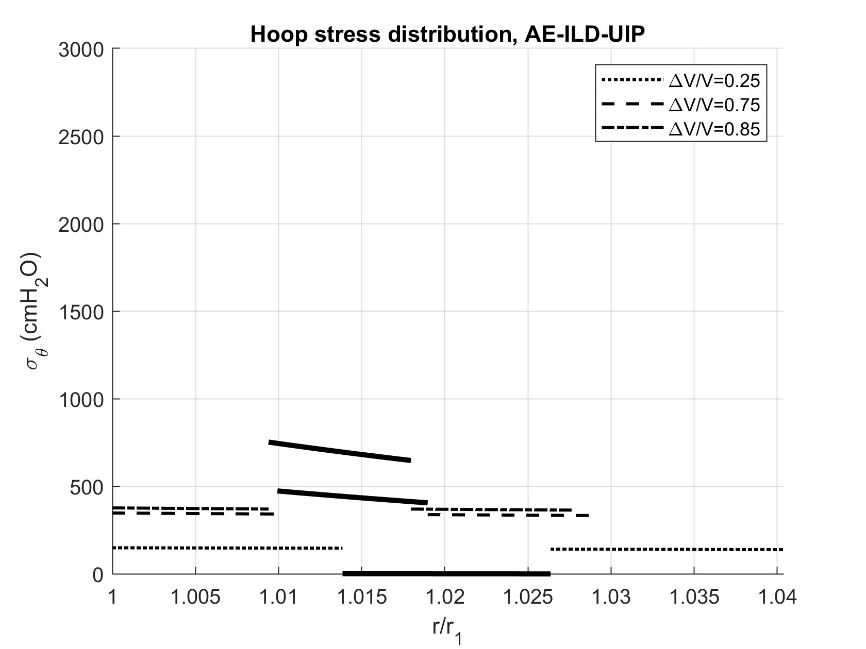

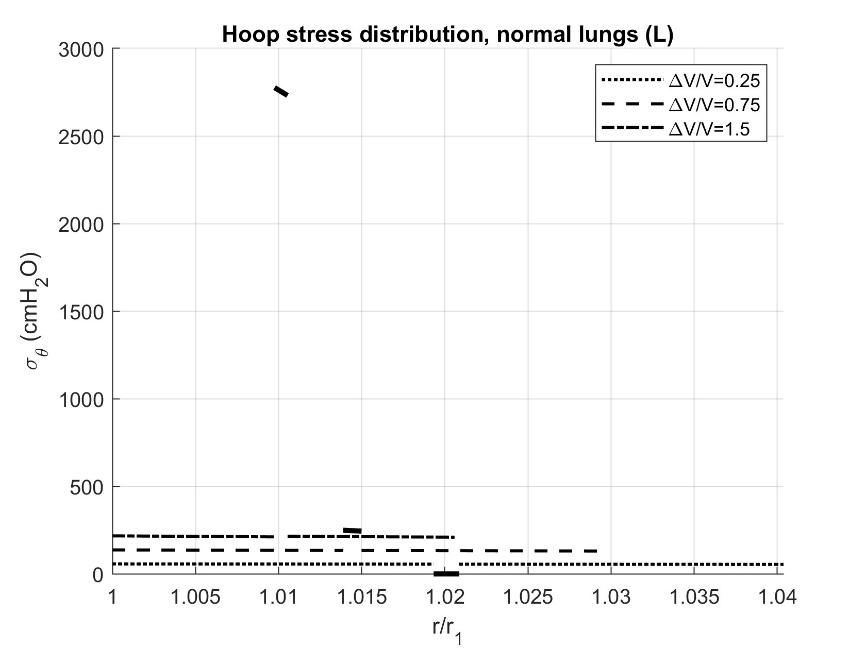

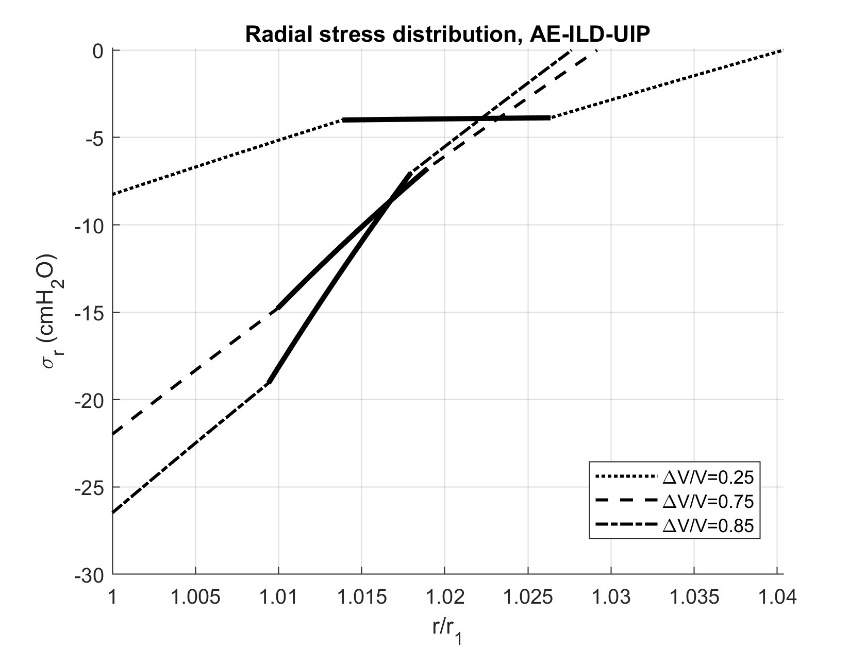

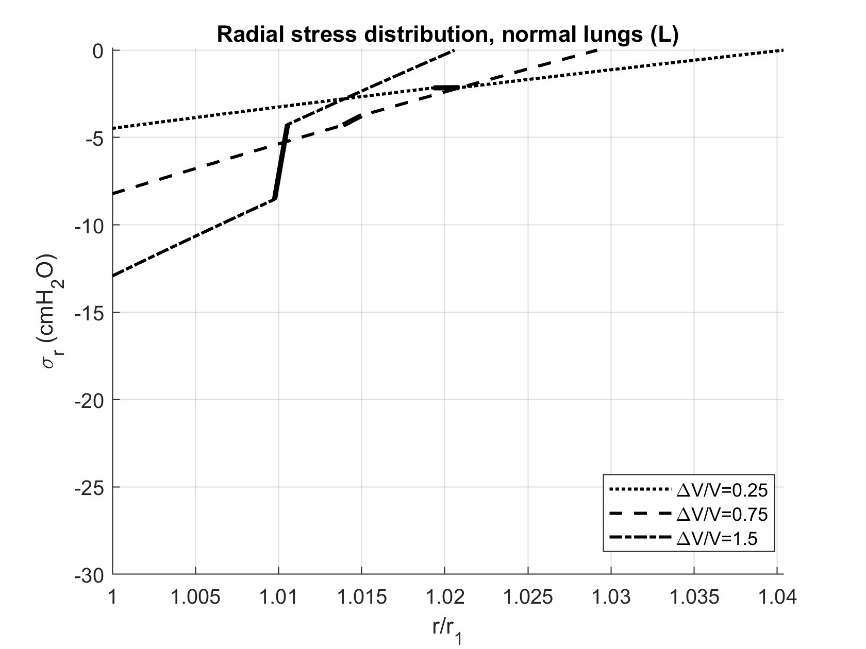


**eFigure 3.** Piecewise distributions of radial and hoop stress in the deformed configuration of a three-layer alveolus, composed of two external layers of elastin and ground material and an inner layer of collagen. Plots on the left column have been obtained using the material parameters estimated from L data (healthy lungs)^14^, plots on the right column using the material parameters estimated from AE-ILD-UIP data (**Table 2**).

*AE-ILD-UIP, acute exacerbation of interstitial lung disease with usual interstitial pneumonia pattern; V=FRC, functional residual capacity.*

**eFigure 4**


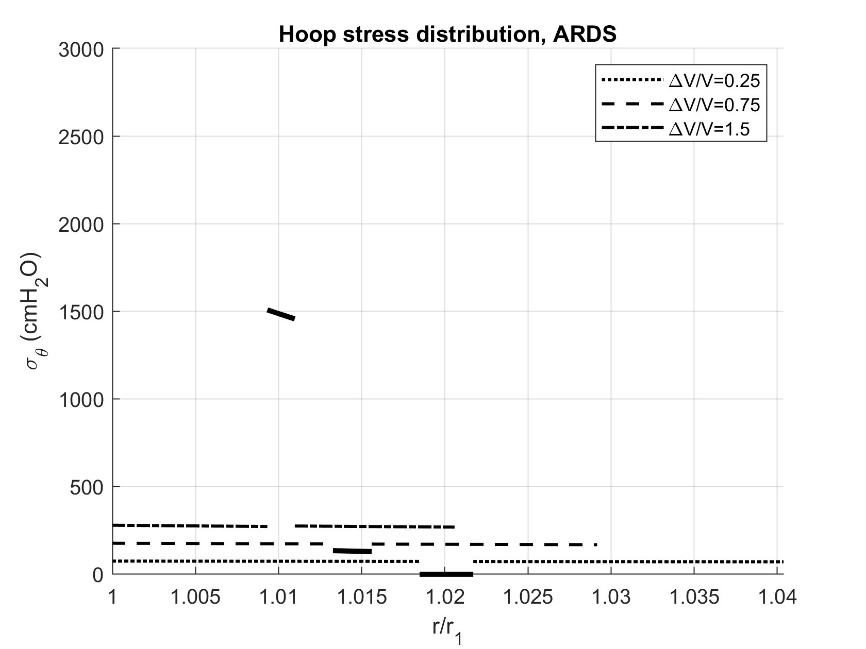

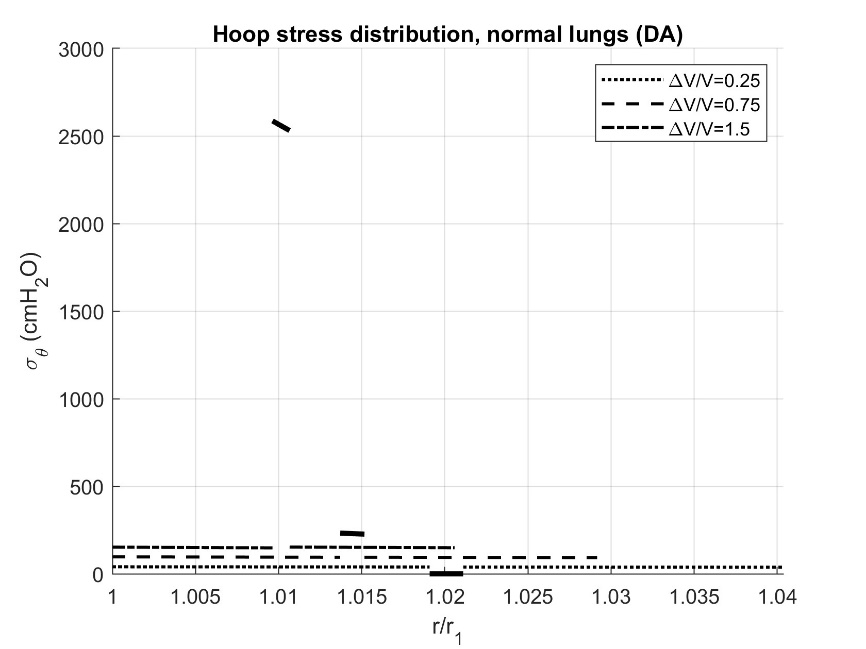

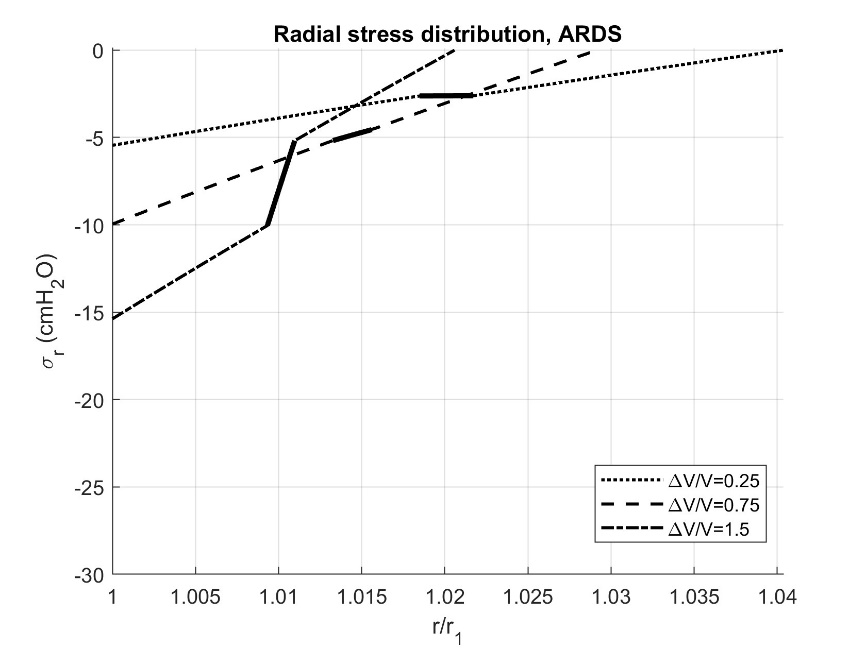

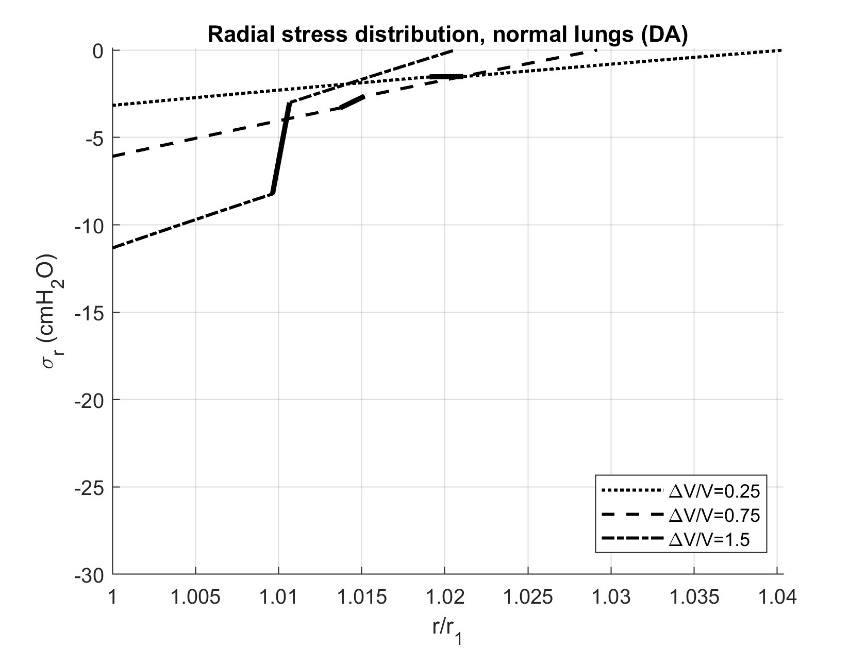


**eFigure 4.** Piecewise distributions of radial and hoop stress in the deformed configuration of a three-layer alveolus, composed of two external layers of elastin and ground material and an inner layer of collagen. Plots on the left column have been obtained using the material parameters estimated from DA data(healthy lungs)^13^, plots on the right column using the material parameters estimated from ARDS data, cf. **Table 2**.

*ARDS, acute respiratory distress syndrome; V=FRC, functional residual capacity.*
