# Supplementary material for: Stress–strain curve and elastic behavior of the fibrotic lung with usual interstitial pneumonia pattern during protective mechanical ventilation
Source: Sci Rep. 2024 Jun 7;14:13158. doi: 10.1038/s41598-024-63670-z (PMC11161630; doi:10.1038/s41598-024-63670-z)
Supplement: Supplementary file 4 — Supplementary Information 4. [file 41598_2024_63670_MOESM4_ESM.docx]

**Supplementary**

**Mechanics variables definition**

End-inspiratory transpulmonary pressure (P_L,EI_) was computed according to the formula

P_L,EI_ = P_plat_ – P_es,EI_

where P_plat_ is end-inspiratory plateau pressure and P_es, EI_ is end-inspiratory esophageal pressure.

Lung stress was defined as P_L,EI_.

The end expiratory lung volume (EELV) measure was based on the nitrogen washout/washin technique through dedicated software (FRC Inview, GE Healthcare). The principle of this technique is as follows: The volume of lung gas includes a volume of nitrogen (V _(1)_ N_2_) that is determined by alveolar fraction of nitrogen (F_A_N_2(1)_) and by the EELV:

V_(1)_N_2_ = F_A_N_2(1)_ X EELV

The alveolar fraction of nitrogen can change by changing the administered FiO2 (F_A_N_2(2)_), resulting in a new volume of nitrogen (V_(2)_N_2_) in the lung after a period of balance.

V_(2)_N_2_ = F_A_N_2(2)_ X EELV

Assuming that after the change of FiO2 the EELV does not change, until a new balance of the alveolar gas is reached, the following equation can be written:

V_(1)_N_2_ – V_(2)_N_2_ = (F_A_N_2(2)_ –FAN_2(1)_) X EELV

As the changes in F_A_N_2_are specular to the changes in FiO_2_ the EELV can be calculated as:

EELV = ΔN_2_(ml)/ΔFiO_2_

where ΔN_2_ is equal to the exhaled nitrogen after the change in FiO_2_ once equilibrium has been reached (20 breaths). The algorithm used by the Engstrom Carestation (FRC Inview, GE Healthcare) employs tidal concentration of oxygen and carbon dioxide to obtain an estimate of nitrogen concentration in expired and inspired air. For more details, see Chiumello et al.^10^ and Olegård et al. ^20^.

Global lung strain was defined according to the formula

Global strain = ΔV/ functional residual capacity (FRC)

where ΔV was computed as follows: EELV – FRC + Vt^5,10^ and FRC is functional residual capacity. FRC was considered as EELV at ZEEP.

Specific elastance was computed at each PEEP level according to the formula

Specific elastance = P_L,EI_ /(ΔV/FRC)

**Equilibrium of a pressurized composite shell**

Consider a spherical shell made of $n$ layers composed of incompressible isotropic hyperelastic materials. Let $X=X\left( R,\Theta,\Phi\right)$ denote the position of a material particle in the undeformed conﬁguration, where the geometry is defined as

$R_{i}\leq R\leq R_{e},$ $0\leq\Theta\leq\pi,$ $0\leq\Phi\leq2\pi,$ (1)

with $R_{i}$ and $R_{e}$ the inner and outer radii of the sphere, respectively. The sphere is divided into $n$ layers, being the $j$-th layer comprised between the radii $R_{j}$ and $R_{j+1}$, $j=1, 2,\ldots n,$ with $R_{1}=R_{i}$ and $R_{n+1}=R_{e}$. Let $h_{j}:=R_{j+1}-R_{j}$ denote the thickness of the $j$-th layer and $\varepsilon_{n+1}:=\frac{h}{R_{i}}$ to denote the (small) ratio of total thickness $h$ to internal radius $R_{i}$.

The multilayered shell is subject to a uniform internal pressure $P$. Let $x=x\left( r,\theta,\varphi\right)$denote the position of a material particle in the deformed conﬁguration and let spherical symmetry be retained during inflation, so that a finite deformation of the shell is given by

$r=f\left( R \right), \theta=\Theta, \varphi=\Phi.$ (2)

The deformed geometry is described by

$r_{1}\leq r\leq r_{n+1},$ $0\leq\theta\leq\pi,$ $0\leq\varphi\leq2\pi.$ (3)

with $r_{1}$ and $r_{n+1}$the inner and outer radii of the deformed conﬁguration, respectively.

The principal stretches are $\lambda_{r}=\frac{dr}{dR}$ in the radial direction and $\lambda_{\theta}=\lambda_{\varphi}=\frac{r}{R}=:\lambda$ in the circumferential directions, respectively. Incompressibility requires that $\lambda_{r}=\lambda^{-2}$ and radial deformation can be calculated as

$f\left( R \right)=\left( R^{3}+r_{1}^{3}-R_{1}^{3} \right)^{\frac{1}{3}}.$ (4)

By continuity, the latter equation holds for $R_{1}\leq R\leq R_{n+1}.$ We also introduce the following notation: $r_{j}:=f\left( R_{j} \right)$, and $\lambda_{j}:=\frac{r_{j}}{R_{j}}$, $j=1, 2,\ldots n,$ being $\lambda_{j}$ the stretches at the inner surfaces between the layers. Using Equation (4), these stretches can be related to the relative volume change $\frac{\Delta V}{V}$ inside the sphere as follows:

$\lambda_{j}=\left( 1+\frac{1}{{(1+\varepsilon_{j})}^{3}}\frac{\Delta V}{V} \right)^{1/3},$ (5)

with $\varepsilon_{j}:=(R_{j}-R_{1})/R_{1}$, $j=1, 2, \ldots n+1.$ Substituting the relation $R_{j}=R_{1}+\sum_{k=1}^{j-1} h_{k}$ into the expression of $\varepsilon_{j}$, we find

$\varepsilon_{j}:=\frac{\sum_{k=1}^{j-1} h_{k}}{R_{1}}=\varepsilon_{n+1}\sum_{k=1}^{j-1} \chi_{k}, j=2,\ldots n+1,$ (6)

with $\chi_{k}:=h_{k}/h$ the fraction of wall thickness of the $k-$th layer. For a very thin shell, the thickness fraction can be approximated by the volume fraction of the $k-$th layer up to a term $O(\varepsilon_{n+1})$. By definition $\varepsilon_{1}=0,$ thus the stretch at the inner surface is simply given by

$\lambda_{1}=\left( 1+\frac{\Delta V}{V} \right)^{1/3}.$ (7)

Note that, in view of Equation. (7), we have $\lambda_{j}>\lambda_{j+1},$ $j =1, 2\ldots n+1.$

The behavior of the material composing the $j$-th layer can be described by the strain energy density $W^{\left( j \right)}\left( \lambda_{r},\lambda_{\theta},\lambda_{\varphi} \right)$, function of $\lambda_{r},\lambda_{\theta},\lambda_{\varphi}$ via the strain invariants. The Cauchy principal stress components in the $j$-th layer are given by

$\sigma_{r}^{\left( j \right)}=\lambda_{r}\frac{\partial W^{\left( j \right)}}{\partial\lambda_{r}}-p^{\left( j \right)}, \sigma_{\theta}^{\left( j \right)}=\sigma_{\varphi}^{\left( j \right)}=\lambda_{\theta}\frac{\partial W^{\left( j \right)}}{\partial\lambda_{\theta}}-p^{\left( j \right)}, j =1, 2\ldots n+1,$ (8)

where $p^{\left( j \right)}, j=1, 2\ldots n+1,$ are arbitrary hydrostatic pressures. Axial symmetry implies that the only nontrivial equilibrium equations are

$\frac{d\sigma_{r}^{\left( j \right)}}{dr}+2\frac{{(\sigma}_{r}^{\left( j \right)}-\sigma_{\theta}^{\left( j \right)})}{r}=0, j =1, 2, \ldots n+1.$ (9)

After introducing the auxiliary functions $w^{\left( j \right)}\left( \lambda\right):=W^{\left( j \right)}\left( \lambda^{-2},\lambda,\lambda\right), j=1, 2, \ldots n+1$, it can be shown that Equations (9) are equivalent to

$\frac{d\sigma_{r}^{\left( j \right)}}{d\lambda}=-\frac{\dot{w}^{\left( j \right)}}{\lambda^{3}-1}, j=1, 2, \ldots n+1,$ (10)

where the dot indicates differentiation with respect to $\lambda$^21^. After integrating and imposing the boundary conditions $\sigma_{r}^{\left( 1 \right)}\left( \lambda_{1} \right)=-P$ and $\sigma_{r}^{\left( N \right)}\left( \lambda_{N+1} \right)=0$, we obtain that

$\sigma_{r}^{\left( 1 \right)}\left( \lambda\right)=\int_{\lambda}^{\lambda_{1}} \frac{\dot{w}^{\left( 1 \right)}\left( l \right)}{l^{3}-1} dl-P, \lambda_{2}\leq\lambda\leq\lambda_{1},$ (11)

$\sigma_{r}^{\left( j \right)}\left( \lambda\right)=\int_{\lambda}^{\lambda_{j}} \frac{\dot{w}^{\left( j \right)}\left( l \right)}{l^{3}-1} dl+\sum_{k=1}^{j-1} \int_{\lambda_{k+1}}^{\lambda_{k}} \frac{\dot{w}^{\left( k \right)}\left( l \right)}{l^{3}-1} dl-P, \lambda_{j+1}\leq\lambda\leq\lambda_{j}, j=2, \ldots n+1,$ (12)

$P=\sum_{j=1}^{n} \int_{\lambda_{j+1}}^{\lambda_{j}} \frac{\dot{w}^{\left( j \right)}\left( l \right)}{l^{3}-1} dl.$ (13)

Equation (13) gives the internal pressure as a function of the stretches $\lambda_{j}$. Finally, given $\sigma_{r}^{\left( j \right)}\left( \lambda\right)$ from Equations (8,9), the circumferential stress can be calculated using the second of Equation. (8) as follows:

$\sigma_{\theta}^{\left( j \right)}\left( \lambda\right)=\frac{1}{2}{\lambda\dot{w}^{\left( j \right)}\left( \lambda\right)+\sigma}_{r}^{\left( j \right)}\left( \lambda\right)$, $\lambda_{j+1}\leq\lambda\leq\lambda_{j},$ $j=1, 2, \ldots n+1.$ (14)

As discussed in^22^, in normal test conditions alveoli can be considered thin shells with a thickness to radius ratio $\varepsilon_{n+1}$close to 0.05. Thus, each $\varepsilon_{j}$ in Equation (11) can be assumed to be a small parameter. Substituting Equations (6) and (5) into Equation (13) and expanding to first order with respect to $\varepsilon_{n+1}$ leads to the following approximation

$P=\varepsilon_{n+1}\sum_{j=1}^{n} \chi_{j}\left( \frac{\dot{w}^{\left( j \right)}(\lambda_{1})}{{\lambda_{1}}^{2}} \right),$ (15)

with $\lambda_{1}$ given by Equation (7). This equation is the formula for inflation pressure for a multilayered balloon. The case of homogenous balloon is considered for example in ^23^ .

*Constitutive modelling of collagen and elastin*

Collagen fibers display a high nonlinear behavior and stiffening at large strains, while elastin and ground substance are softer. A simple choice for their strain energy densities is

$W^{\left( 1 \right)}\left( \lambda_{r},\lambda_{\theta},\lambda_{\varphi} \right)=c_{1}\left( \lambda_{r}^{2}+\lambda_{\theta}^{2}+\lambda_{\varphi}^{2}-3 \right),$ (16)

$W^{\left( 2 \right)}\left( \lambda_{r},\lambda_{\theta},\lambda_{\varphi} \right)=c_{2}\left( \lambda_{r}^{2}+\lambda_{\theta}^{2}+\lambda_{\varphi}^{2}-3 \right)^{3},$ (17)

with $c_{1}, c_{2}$ positive material constants. Following the previously introduced notation, in Equations (16, 17), the index 1 stands for material of the first layer, that is composed for elastin, while the index 2 stands for material of the second layer, composed of collagen. In the remaining of the paper, we will assume that the layers with odd index are composed of the same material of layer 1 (elastin) and layers with even index are composed of the same material of layer 2 (collagen), in such a way that $W^{\left( i \right)}=W^{\left( 1 \right)}$ for $i=3, 5, 7,\ldots$, and $W^{\left( i \right)}=W^{\left( 2 \right)}$ for $i=2, 4, 6,\ldots$.

The choice provided by Equation (16,17) agrees with the constitutive model of lung parenchyma experimentally determined via uniaxial tension tests on living precision-cut rat lung slices ^24^**^,^** ^25^. The main difference between Equations (16), (17) and the constitutive model proposed by Rausch and coworkers ^24^ is that here we assume the materials to be incompressible, while they account for an energy term to control the volumetric change. We also note that the stress-strain response in traction of the constitutive model (17) can be interpreted as a phenomenological description of the toe region followed by gradual, nonlinear stiffening of curly collagen fibers, due to progressive recruitment of fibers characterized by different amounts of slackness^26^. For multiscale modeling of collagen and proteins with unfolding domains the reader is referred to the literature^22^**^,^** ^26^**^,^** ^27^**^,^** ^28^**^,^** ^29^. It is worth mentioning that while the material constant $c_{1}$ is proportional to the initial elasticity modulus of elastin in traction test, the material constant $c_{2}$ can not be thought to represent such a quantity for collagen. Indeed, the stress-strain relation in traction corresponding to the energy given by Equation (17) presents a vanishing slope at the origin, corresponding to zero initial elasticity modulus. So, an increase of $c_{2}$ can be more appropriately interpreted as an increase of collagen stiffening occurring at larger strains.

**Pressure-volume relation**

Even though the present model allows for the description of multiple layers of collagen alternated to layers composed of elastin and ground material, for computational testing we assume the alveolus made of only three layers, the two external ones composed of elastin and ground material, and one in between composed of collagen.

We take$\chi_{2}$ to denote the thickness fraction of collagen, and $1-\chi_{2}$ the remaining fraction of elastin and ground substance. With this notation, the pressure-volume for the shell model can be obtained by substituting Equations (16,17) into Equation (13). We find that

$P=\mathcal{-g}_{1}\left( \lambda_{2} \right)+\mathcal{g}_{1}\left( \lambda_{1} \right)-\mathcal{g}_{2}\left( \lambda_{3} \right)+\mathcal{g}_{2}\left( \lambda_{2} \right)-\mathcal{g}_{1}\left( \lambda_{4} \right)+\mathcal{g}_{1}\left( \lambda_{3} \right),$ (18)

with

$\mathcal{g}_{1}\left( z \right):= \int\frac{\dot{w}^{\left( 1 \right)}\left( l \right)}{l^{3}-1} dl=-\frac{c_{1}}{z^{4}}-\frac{4c_{1}}{z}$, (19)

$\mathcal{g}_{2}\left( z \right):= \int\frac{\dot{w}^{\left( 2 \right)}\left( l \right)}{l^{3}-1} dl=3c_{2}(-\frac{1}{3z^{12}}-\frac{4}{9z^{9}}+\frac{3}{z^{8}}-\frac{8}{3z^{6}}+\frac{24}{5z^{5}}-\frac{9}{z^{4}}-\frac{16}{3z^{3}}+\frac{24}{z^{2}}-\frac{36}{z}-48z+\frac{16z^{3}}{3}+16\ln z)$.

(20)

In Equation (18), the stretches $\lambda_{j}, j=1,2,3,4,$ defined by Equation (5), can be more conveniently rewritten in terms of $\chi_{2}$ and $\frac{\Delta V}{V}$as follows:

$\lambda_{2}=\left( 1+\frac{1}{\left( 1+\varepsilon_{n+1}{(1-\chi}_{2})/2 \right)^{3}}\frac{\Delta V}{V} \right)^{\frac{1}{3}},$ (21)

$\lambda_{3}=\left( 1+\frac{1}{\left( 1+\varepsilon_{n+1}{(1+\chi}_{2})/2 \right)^{3}}\frac{\Delta V}{V} \right)^{\frac{1}{3}},$ (22)

$\lambda_{4}=\left( 1+\frac{1}{\left( 1+\varepsilon_{n+1} \right)^{3}}\frac{\Delta V}{V} \right)^{\frac{1}{3}}.$ (23)

Notably, the isolated contributions of elastin (and ground substance) and collagen in Equation (18) are given by the terms containing the functions $\mathcal{g}_{1}$ and $\mathcal{g}_{2}$, respectively.

**Radial and hoop stress distributions**

For the three layers configuration constituted of a layer of collagen interposed between two outermost layers of elastin and ground material, the piecewise distribution of radial stress along the thickness of the deformed shell can be obtained by substituting Equations (16,17) into (11,12) to get the following relation:

$\left\{ \begin{matrix} \sigma_{r}^{\left( 1 \right)}\left( \frac{r}{r_{1}} \right)=-P+\mathcal{g}_{1}\left( \mathcal{l}\left( \frac{r}{r_{1}} \right) \right)-\mathcal{g}_{1}\left( \lambda_{1} \right), \zeta_{1}\leq\frac{r}{r_{1}}\leq\zeta_{2}, \\ \sigma_{r}^{\left( 2 \right)}\left( \frac{r}{r_{1}} \right)=-P+\mathcal{g}_{1}\left( \lambda_{2} \right)-\mathcal{g}_{1}\left( \lambda_{1} \right)+\mathcal{g}_{2}\left( \mathcal{l}\left( \frac{r}{r_{1}} \right) \right)-\mathcal{g}_{2}\left( \lambda_{2} \right), \zeta_{2}<\frac{r}{r_{1}}\leq\zeta_{3}, \\ \sigma_{r}^{\left( 3 \right)}\left( \frac{r}{r_{1}} \right)=-P+\mathcal{g}_{1}\left( \lambda_{2} \right)-\mathcal{g}_{1}\left( \lambda_{1} \right)+\mathcal{g}_{2}\left( \lambda_{3} \right)-\mathcal{g}_{2}\left( \lambda_{2} \right)+\mathcal{g}_{1}\left( \mathcal{l}\left( \frac{r}{r_{1}} \right) \right)-\mathcal{g}_{1}\left( \lambda_{3} \right), \zeta_{3}<\frac{r}{r_{1}}\leq\zeta_{4}, \end{matrix} \right.$ (24)

with

$\mathcal{l}\left( z \right)=\left( \frac{z^{3}}{z^{3}-\frac{\frac{\Delta V}{V}}{1+\frac{\Delta V}{V}}} \right)^{\frac{1}{3}},$ (25)

and $\zeta_{j}:=\frac{r_{j}}{r_{1}}, j=1,2,3,4,$ the ratios between the deformed radius $r_{j}$ at the surfaces between the layers and the inner deformed radius $r_{1}.$ Thus $\zeta_{1}=1,$ while $\zeta_{j}, j=2,3,4$ take the following form in terms of $\chi_{2}$ and $\frac{\Delta V}{V}:$

$\zeta_{1}=\left( \frac{\frac{\Delta V}{V}}{1+\frac{\Delta V}{V}}+\frac{\left( 1+\varepsilon_{n+1}{(1-\chi}_{2})/2 \right)^{3}}{1+\frac{\Delta V}{V}} \right)^{\frac{1}{3}},$ (26)

$\zeta_{2}=\left( \frac{\frac{\Delta V}{V}}{1+\frac{\Delta V}{V}}+\frac{\left( 1+\varepsilon_{n+1}{(1+\chi}_{2})/2 \right)^{3}}{1+\frac{\Delta V}{V}} \right)^{\frac{1}{3}},$ (27)

$\zeta_{3}=\left( \frac{\frac{\Delta V}{V}}{1+\frac{\Delta V}{V}}+\frac{\left( 1+\varepsilon_{n+1} \right)^{3}}{1+\frac{\Delta V}{V}} \right)^{\frac{1}{3}}.$ (28)

From Equation (24), using Equation (14), the piecewise distribution of hoop stress along the thickness of the deformed shell can be shown to take the following expression:

$\left\{ \begin{matrix} \sigma_{\theta}^{\left( 1 \right)}\left( \frac{r}{r_{1}} \right)=\frac{1}{2}\mathcal{l}\left( \frac{r}{r_{1}} \right) \dot{w}^{\left( 1 \right)}\left( \mathcal{l}\left( \frac{r}{r_{1}} \right) \right)+\sigma_{r}^{\left( 1 \right)}\left( \frac{r}{r_{1}} \right), \zeta_{1}\leq\frac{r}{r_{1}}\leq\zeta_{2}, \\ \sigma_{\theta}^{\left( 2 \right)}\left( \frac{r}{r_{1}} \right)=\frac{1}{2}\mathcal{l}\left( \frac{r}{r_{1}} \right) \dot{w}^{\left( 2 \right)}\left( \mathcal{l}\left( \frac{r}{r_{1}} \right) \right)+\sigma_{r}^{\left( 2 \right)}\left( \frac{r}{r_{1}} \right), \zeta_{2}<\frac{r}{r_{1}}\leq\zeta_{3}, \\ \sigma_{\theta}^{\left( 3 \right)}\left( \frac{r}{r_{1}} \right)=\frac{1}{2}\mathcal{l}\left( \frac{r}{r_{1}} \right) \dot{w}^{\left( 1 \right)}\left( \mathcal{l}\left( \frac{r}{r_{1}} \right) \right)+\sigma_{r}^{\left( 3 \right)}\left( \frac{r}{r_{1}} \right), \zeta_{3}<\frac{r}{r_{1}}\leq\zeta_{4}. \end{matrix} \right.$ (29)

**eFigure 1**

**
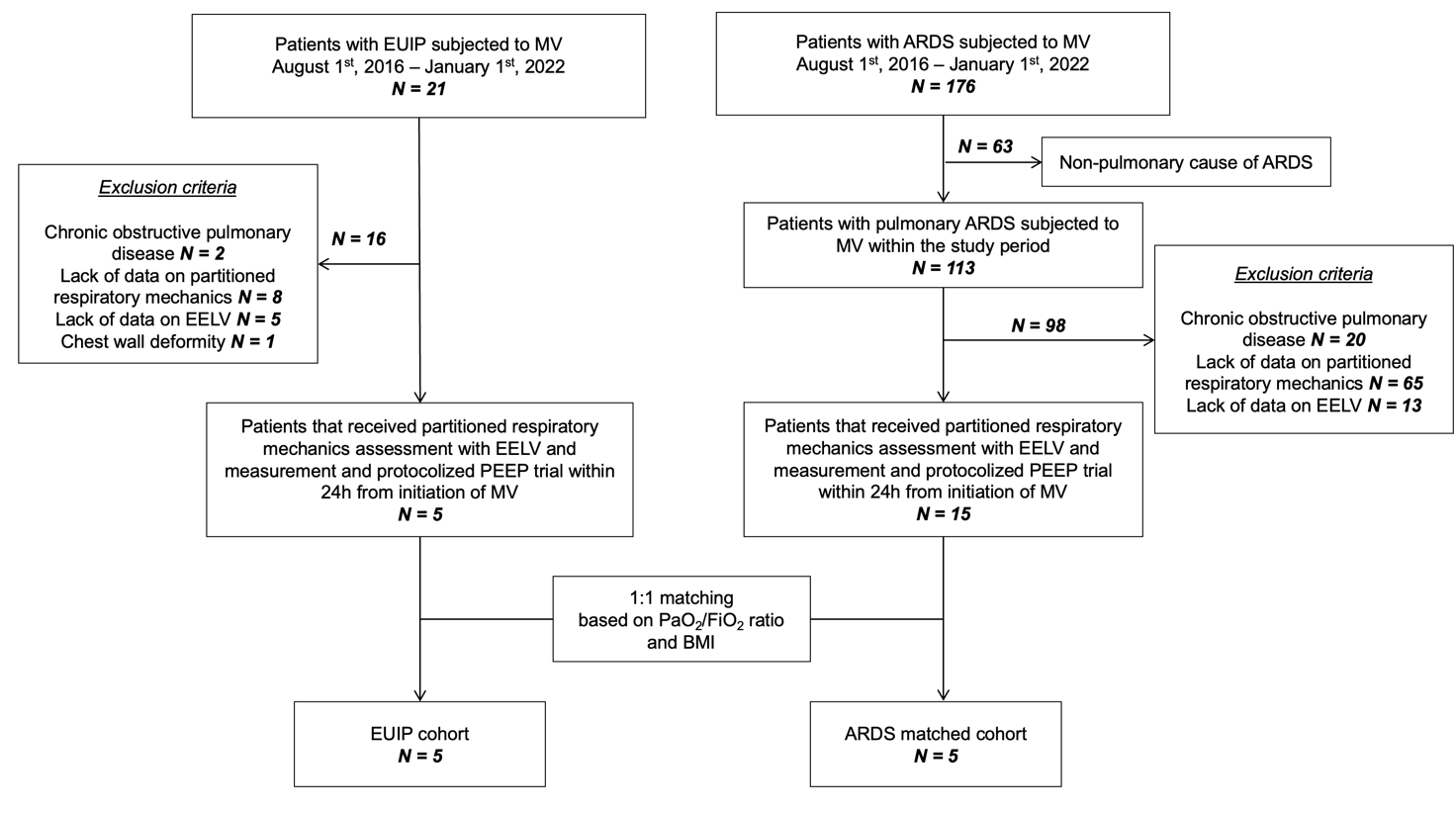
**

**eFigure 1.** Study flow diagram.

*EUIP, acute exacerbation of interstitial lung disease with usual interstitial pneumonia pattern; ARDS, acute respiratory distress syndrome; MV, mechanical ventilation; PEEP, positive end-expiratory pressure; EELV end-expiratory lung volume; BMI, body mass index*

**eTable 1**

| Parameter | EUIP  (n=5) | ARDS  (n=5) | p value |
| --- | --- | --- | --- |
| **Age, years** | 62 (56 – 68) | 64 (53 – 70) | 0.9 |
| **Male, n** | 4 (80) | 4 (80) | 0.9 |
| **BMI, kg/m^2^** | 22.3 (20.8 – 25) | 22.4 (20.8 – 23.8) | 0.8 |
| **Height, m** | 1.77 (1.71 – 1.84) | 1.83 (1.79 – 1.85) | 0.2 |
| **Charlson index, score** | 4 (3 – 5) | 4 (3 – 5) | 0.9 |
| **APACHE, score** | 13 (11.5 – 14) | 14 (12.5 – 14) | 0.7 |
| **SAPS II, score** | 27 (24.5 – 32.5) | 31 (24 – 32) | 0.6 |
| **†PaO_2_/FiO_2_, mmHg** | 90 (77 – 115) | 85 (73 – 111) | 0.8 |
| **RICU/ICU survival, n** | 0 (0) | 3 (60) | 0.01 |

**eTable 1.** General and clinical characteristics in the study groups on admission. Data are presented as number (n) and percentage for dichotomous values or median and IQR for continuous values.

† The values of PaO_2_/FiO_2_ ratio used for matching these groups were those measured at the time of RICU or ICU admission

*EUIP, acute exacerbation of interstitial lung disease with usual interstitial pneumonia pattern; ARDS, acute respiratory distress syndrome; BMI, body mass index; APACHE II, Acute Physiology and Chronic Health Evaluation II; SAPS II; Simplified Acute Physiology Score; RICU, respiratory intensive care unit; ICU, intensive care unit; IQR, interquartile range*

**eFigure 2**

**eFigure 2.** Measured individual values of strain in the matched study groups at ZEEP, PEEP_LOW_ and PEEP_TITRATED_ phase. When testing as an interaction for whether the change in strain at incremental PEEP value was different between EUIP and ARDS, statistical difference was found (p=0.003).

*ZEEP, zero positive end-expiratory pressure; PEEP, positive end-expiratory pressure; EUIP, acute exacerbation of interstitial lung disease with usual interstitial pneumonia pattern; ARDS, acute respiratory distress syndrome*

**Mechanical considerations on stress distribution**

**eFigure 3** and **eFigure 4** show the piecewise distributions of the radial and hoop stress components, $\sigma_{r}$ and $\sigma_{\theta}$ respectively, in three deformed (inflated) configurations of the alveolus, corresponding to three different values of the relative volume change $\frac{\Delta V}{V}$, with V=FRC. The plots have been obtained by inserting the values of the material parameters listed in **Table 2** into Equations (24-29). For normal lungs (DA and L data) and ARDS lungs, the following values of the relative volume change have been considered: $\frac{\Delta V}{V}=0.25, 0.75$ and $1.5$. For AE-ILD-UIP lungs, characterized by a steeper pressure-volume response, the distributions have been plotted for $\frac{\Delta V}{V}=0.25, 0.75$ and $0.85.$ In **eFigure 3** and **eFigure 4**, the stress components $\sigma_{r}$ and $\sigma_{\theta}$ have been represented versus the normalized radial position in the deformed configuration, $\frac{r}{r_{1}},$ with $r$ the radial position and $r_{1}$ the (deformed) radius of the internal shell surface. The normalized radial position ranges from 1 to $\zeta_{4},$ with $r_{4}=\zeta_{4} r_{1}$ the (deformed) radius of the external shell surface. As $\frac{\Delta V}{V}$ increases the shell gets thinner, and correspondingly $\zeta_{4}$ and the domain on the horizontal axis shrink.

Equilibrium at the interfaces between the layers implies the stress component $\sigma_{r}$ to be a continuous function of $\frac{r}{r_{1}}$. Equilibrium at the boundary implies $\sigma_{r}$ to equal $-P$ at the inner surface and to vanish at the outer surface.

The discontinuity of the stress component $\sigma_{\theta}$ along the sphere thickness, i.e., with respect to $\frac{r}{r_{1}},$ is due to the inhomogeneity of the material. The plots on the bottom of **eFigure 3** and **eFigure 4** show that $\sigma_{\theta}$ takes values up to two orders of magnitude greater than $\sigma_{r}.$ For a homogeneous alveolus, this effect would be due to equilibrium, as schematically represented on the right-hand side of Figure 1, where the case of a homogeneous sphere is shown. Considering for simplicity the case of a very thin shell (a balloon) of radius $R$ and thickness $h$, force equilibrium requires ${2\pi\mathrm{Rh}\sigma}_{\theta}=\pi R^{2}P,$ implying $\sigma_{\theta}=\frac{\mathrm{RP}}{2h}.$For a ratio $h/R$ of the order of 0.05 (like in normal lungs, see Jawde et al. ^22^), $\sigma_{\theta}$ turns out to be one order of magnitude greater than the pressure $P.$ The internal collagen layer, undergoing increasing stiffening for large strains, withstands an even larger stress, that can reach over two orders of magnitude the value of the internal pressure, as indicated by the distribution of hoop stress for the largest values $\frac{\Delta V}{V}$in the plots on the bottom of **eFigure 3** and **eFigure 4.**

**eFigure 3**


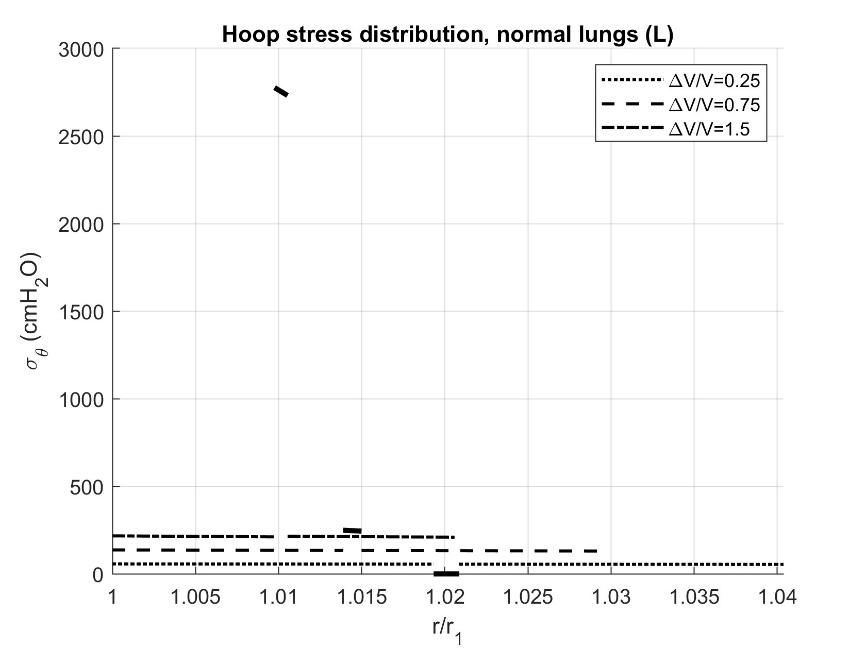

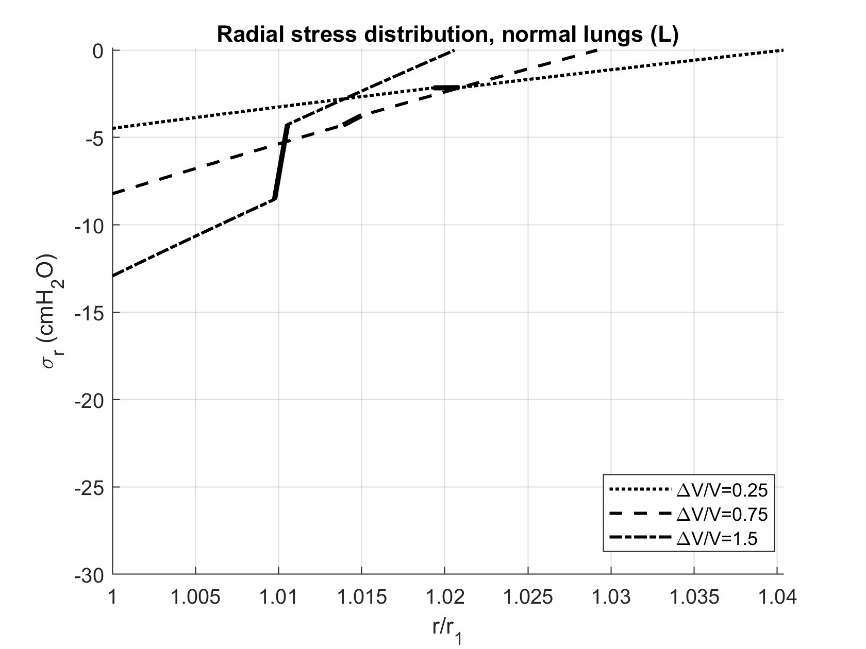

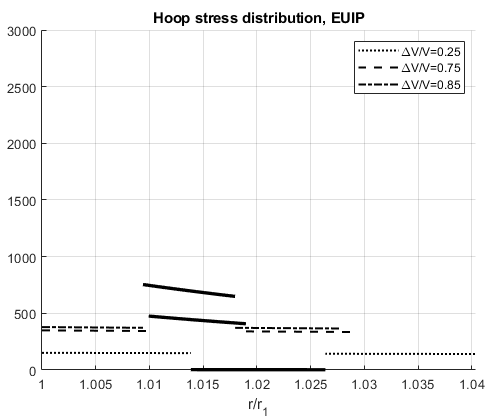

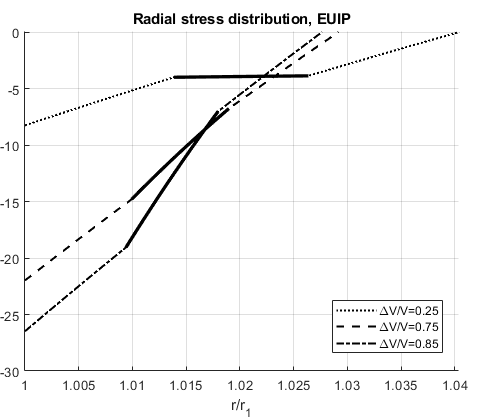


**eFigure 3.** Piecewise distributions of radial and hoop stress in the deformed configuration of a three-layer alveolus, composed of two external layers of elastin and ground material and an inner layer of collagen. Black thick lines correspond to the collagen layer while the other lines correspond to the elastin and ground substance layers. Plots on the left column have been obtained using the material parameters estimated from L data (healthy lungs)^15^, plots on the right column using the material parameters estimated from EUIP data (**Table 2**).

*EUIP, acute exacerbation of interstitial lung disease with usual interstitial pneumonia pattern; V=FRC, functional residual capacity.*

**eFigure 4**


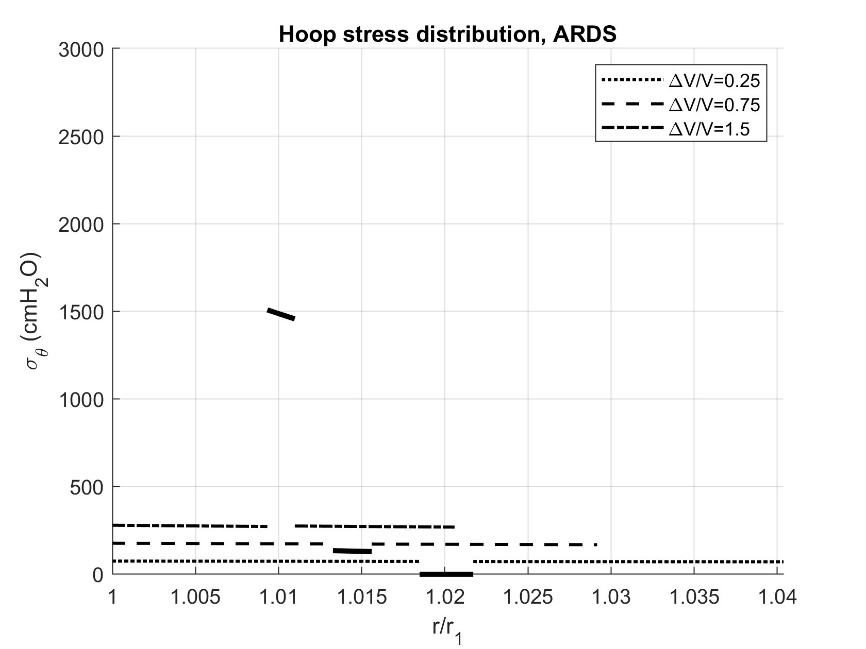

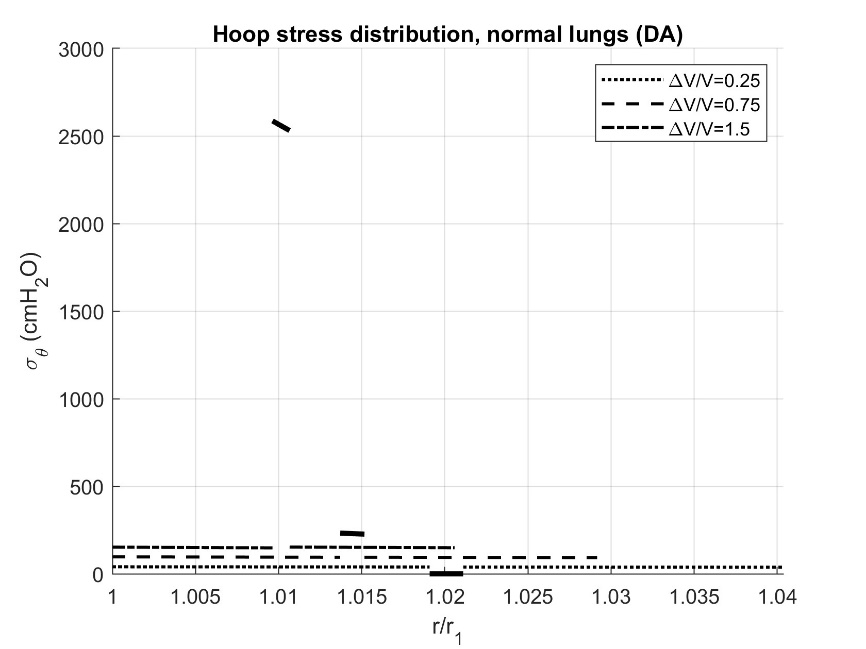

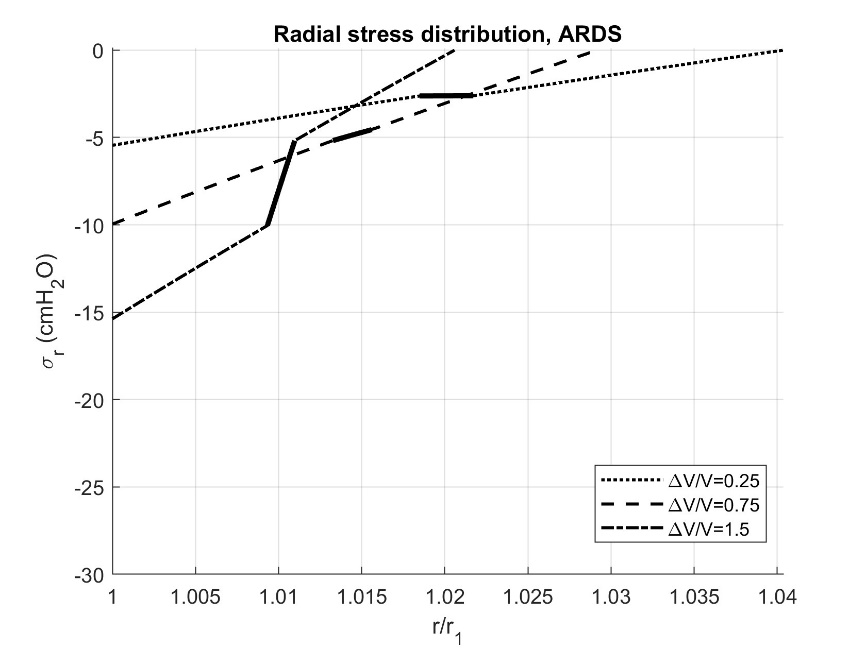

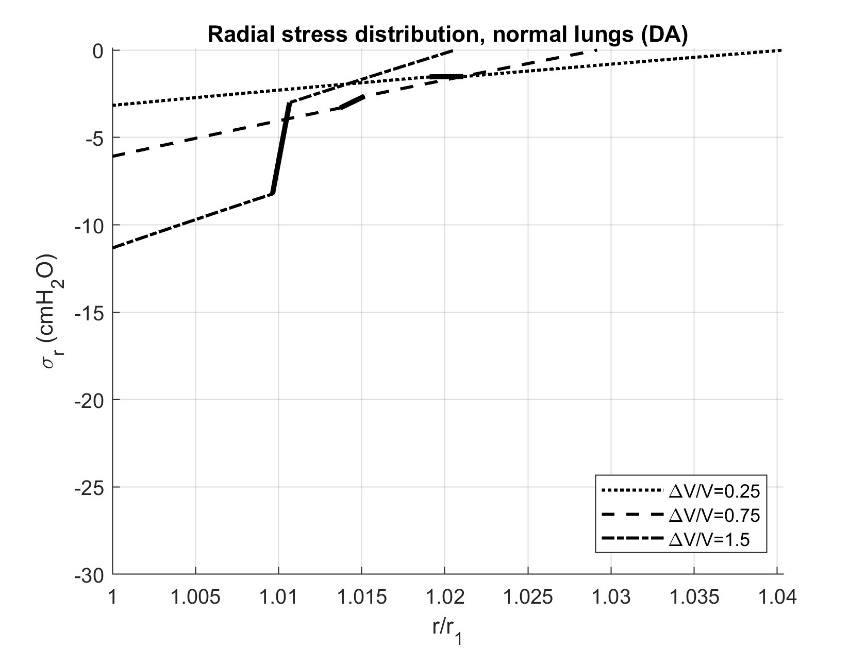


**eFigure 4.** Piecewise distributions of radial and hoop stress in the deformed configuration of a three-layer alveolus, composed of two external layers of elastin and ground material and an inner layer of collagen. Black thick lines correspond to the collagen layer while the other lines correspond to the elastin and ground substance layers. Plots on the left column have been obtained using the material parameters estimated from DA data (healthy lungs)^14^, plots on the right column using the material parameters estimated from ARDS data, cf. **Table 2**.

*ARDS, acute respiratory distress syndrome; V=FRC, functional residual capacity.*

**References**

5. Gattinoni, L., Tonetti, T. & Quintel, M. Regional physiology of ARDS. *Crit. Care* 21, 312 (2017).

10. Chiumello, D. *et al.* Lung Stress and Strain during Mechanical Ventilation for Acute Respiratory Distress Syndrome. *Am. J. Respir. Crit. Care Med.* 178, 346–355 (2008).

20. Olegård, C., Söndergaard, S., Houltz, E., Lundin, S. & Stenqvist, O. Estimation of Functional Residual Capacity at the Bedside Using Standard Monitoring Equipment: A Modified Nitrogen Washout/Washin Technique Requiring a Small Change of the Inspired Oxygen Fraction: *Anesth. Analg.* 101, 206–212 (2005).

14. D’Angelo, E. et al. Respiratory mechanics in anesthetized paralyzed humans: effects of flow, volume, and time. J. Appl. Physiol. 67, 2556–2564 (1989).

15. Levy, P. et al. A method for studying the static volume-pressure curves of the respiratory system during mechanical ventilation. J. Crit. Care 4, 83–89 (1989).

21. deBotton, G., Bustamante, R. & Dorfmann, A. Axisymmetric bifurcations of thick spherical shells under inflation and compression. Int. J. Solids Struct. 50, 403–413 (2013).

22. Jawde, S. B. et al. Inflation instability in the lung: an analytical model of a thick-walled alveolus with wavy fibres under large deformations. J. R. Soc. Interface 18, 20210594 (2021).

23. Beatty, M. F. Topics in Finite Elasticity: Hyperelasticity of Rubber, Elastomers, and Biological Tissues—With Examples. Appl. Mech. Rev. 40, 1699–1734 (1987).

24. Rausch, S. M. K., Martin, C., Bornemann, P. B., Uhlig, S. & Wall, W. A. Material model of lung parenchyma based on living precision-cut lung slice testing. J. Mech. Behav. Biomed. Mater. 4, 583–592 (2011).

25. Birzle A.M., Hobrack S.M.K., Martin C., Uhlig S., Wall A.W. Constituent-specific material behavior of soft biological tissue: experimental quantification and numerical identification for lung parenchyma. Biomech Model Mechanobiol 2019; 18: 1383-1400.

26. Fratzl, P. et al. Fibrillar Structure and Mechanical Properties of Collagen. J. Struct. Biol. 122, 119–122 (1998).

27. Marino, M. & Vairo, G. Multiscale Elastic Models of Collagen Bio-structures: From Cross-Linked Molecules to Soft Tissues. in Multiscale Computer Modeling in Biomechanics and Biomedical Engineering (ed. Gefen, A.) vol. 14 73–102 (Springer Berlin Heidelberg, Berlin, Heidelberg, 2013).

28. De Tommasi, D., Puglisi, G. & Saccomandi, G. Multiscale mechanics of macromolecular materials with unfolding domains. J. Mech. Phys. Solids 78, 154–172 (2015).

29. Puglisi, G., De Tommasi, D., Pantano, M. F., Pugno, N. M. & Saccomandi, G. Micromechanical model for protein materials: From macromolecules to macroscopic fibers. Phys. Rev. E 96, 042407 (2017).
